# Comammox *Nitrospira* act as key bacteria in weakly acidic soil via potential cobalamin sharing

**DOI:** 10.1002/imt2.271

**Published:** 2025-02-04

**Authors:** Yuxiang Zhao, Jiajie Hu, Jiaqi Wang, Xiangwu Yao, Tong Zhang, Baolan Hu

**Affiliations:** ^1^ Key Laboratory of Environment Remediation and Ecological Health, Ministry of Education, College of Environmental Resource Sciences Zhejiang University Hangzhou China; ^2^ College of Environmental and Resource Sciences Zhejiang University Hangzhou China; ^3^ Environmental Microbiome Engineering and Biotechnology Laboratory, Department of Civil Engineering The University of Hong Kong Hong Kong SAR China; ^4^ School of Public Health The University of Hong Kong Hong Kong SAR China; ^5^ Center for Environmental Engineering Research The University of Hong Kong Hong Kong SAR China; ^6^ Zhejiang Province Key Laboratory for Water Pollution Control and Environmental Safety Hangzhou China

**Keywords:** acidic soils, bacterial interaction, cobalamin, comammox *Nitrospira*

## Abstract

The discovery of comammox *Nitrospira* in low pH environments has reshaped the ammonia oxidation process in acidic settings, providing a plausible explanation for the higher nitrification rates observed in weakly acidic soils. However, the response of comammox *Nitrospira* to varying pH levels and its ecological role in these environments remains unclear. Here, a survey across soils with varying pH values (ranging from 4.4 to 9.7) was conducted to assess how comammox *Nitrospira* perform under different pH conditions. Results showed that comammox *Nitrospira* dominate ammonia oxidation in weakly acidic soils, functioning as a K‐strategy species characterized by slow growth and stress tolerance. As a key species in this environment, comammox *Nitrospira* may promote bacterial cooperation under low pH conditions. Genomic evidence suggested that cobalamin sharing is a potential mechanism, as comammox *Nitrospira* uniquely encode a metabolic pathway that compensates for cobalamin imbalance in weakly acidic soils, where 86.8% of metagenome‐assembled genomes (MAGs) encode cobalamin‐dependent genes. Additionally, we used DNA stable‐isotope probing (DNA‐SIP) to demonstrate its response to pH fluctuations to reflect how it responds to the decrease in pH. Results confirmed that comammox *Nitrospira* became dominant ammonia oxidizers in the soil after the decrease in pH. We suggested that comammox *Nitrospira* will become increasingly important in global soils, under the trend of soil acidification. Overall, our work provides insights that how comammox *Nitrospira* perform in weakly acidic soil and its response to pH changes.

## Highlights

- Comammox *Nitrospira* are dominant ammonia oxidizers in weakly acidic soil.
- Comammox *Nitrospira* are the K‐strategy species in weakly acidic soil.
- Comammox *Nitrospira* may promote bacterial cooperation under low pH conditions via cobalamin sharing.

## Graphical abstract

Comammox *Nitrospira* are the dominant ammonia oxidizers in weakly acidic soil. They are the K‐strategy species in weakly acidic soil and may promote bacterial cooperation under low pH conditions via cobalamin sharing.

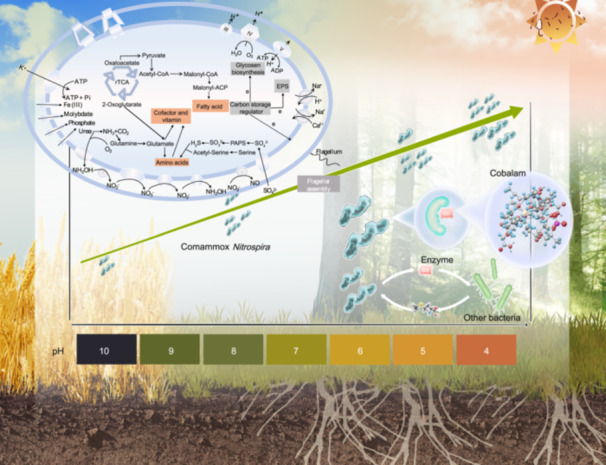

## INTRODUCTION

Nitrification is an essential step of the biogeochemical nitrogen (N) cycle, which could convert ammonia to nitrate via nitrite [1]. Ammonia‐oxidizing microorganisms (AOMs), key drivers of nitrification, contribute to the oxidation of ~2330 Tg N/year, representing one of the largest nitrogen fluxes in the global nitrogen budget [2]. Traditionally, chemolithoautotrophic nitrification has been considered a two‐step process, with ammonia‐oxidizing bacteria (AOB) and archaea (AOA) responsible for oxidizing ammonia to nitrite [3], followed by nitrite‐oxidizing bacteria, that convert nitrite to nitrate.

Environmental conditions are key factors driving the niche differentiation of terrestrial AOMs [4], with pH considered as the primary determinant [5]. Low pH adversely affects nitrification due to its constraints on free ammonia (the actual substrate for AOMs) and elevated proton concentrations [6]. However, some controversy has emerged. According to a meta‐analysis by Booth et al., there is a significant negative relationship between terrestrial nitrification and pH, with some of the fastest nitrification rates occurring at pH = 5 [7]. This may be because several AOA (*Ca*. Nitrosotalea) and AOB (*Ca*. Nitrosacidococcus and *Ca*. Nitrosoglobus terrae TAO100 [8, 9, 10]) thrive in acidic environments. These acidophilic or acid‐tolerant ammonia oxidizers have a common feature in their typically higher affinity for ammonia, ranging from (0.6 to 42 nM‐NH_3_) [8, 9, 10]. However, considering the weakly negative correlation between AOA abundance and pH at the macro‐scale [11, 12], as well as the similarity with AOB abundance in several acidic soils, AOA does not solely dominate all acidic soils. Thus, we posited the existence of another AOM that could potentially dominate the ammonia oxidation process in acidic soils.

In 2015, a novel AOM, comammox *Nitrospira*, was discovered, which can oxidize ammonia to nitrate in a single cell, which has radically changed the two‐step nitrification theory [13, 14]. Comammox *Nitrospira* belongs to lineage II of the genus *Nitrospira*, which was previously considered to only perform nitrite oxidation [15]. Notably, comammox *Nitrospira* exhibit a markedly higher apparent ammonia affinity (0.06–0.08 μM) compared to traditional AOB and the majority of AOA [16, 17], potentially aiding its acclimatization to weakly acidic conditions. Based on a meta‐analysis of all habitats, Zhu et al. suggested that pH is the main factor influencing the abundance of comammox *Nitrospira* [18]. Li et al. enriched comammox *Nitrospira* in a weakly acidic bioreactor [19], and discovered novel strains in an acid mine lake [20], suggesting that comammox *Nitrospira* may dominate ammonia oxidation in acidic waters. The presence of comammox *Nitrospira* in acidic soils has also been suggested in various land use types, including orchards, forests, and farmland [21, 22]. However, how pH influences comammox *Nitrospira* remain unclear.

pH not only influences the composition of microbial communities but also alters microbial interactions [23]. Microbial life is mostly competitive [24]. However, Zhao et al. suggested that under stressful conditions, microbial cooperation based on public goods sharing becomes the dominant interaction [25]. This observation aligns with the stress gradient hypothesis (SGH), which predicts that competition prevails in permissive environments while abiotic stress fosters more positive interactions [26]. Under environmental stress, comammox *Nitrospira* exhibit cooperative behavior with other microbes, including forming biofilms to withstand high dissolved oxygen and ammonia concentrations [27], as well as establishing symbiotic associations with anammox bacteria through the exchange of nitrite and formate [28]. Low pH undoubtedly acts as a stress for microorganisms, potentially enhancing microbial cooperation. Given that comammox *Nitrospira* are hypothesized to be the dominant ammonia oxidizers in weakly acidic soils based on their apparent ammonia affinity, it is crucial to investigate their ecological role and contribution to microbial interactions.

Herein, we revealed the relationship between comammox *Nitrospira* and pH with multiple metagenomic approaches and analyses. Moreover, we reconstructed the metagenome‐assembled genomes (MAGs) of comammox *Nitrospira* to reveal its metabolic potential and confirmed the genomic findings with DNA stable‐isotope probing (DNA‐SIP). Specifically, we aimed to address three areas: (i) How pH influences comammox *Nitrospira*? (ii) What is its ecological status in weakly acidic soil? (iii) Why it can dominate the nitrification in weakly acidic soil?

## RESULTS

### Changes in the abundance and activity of ammonia oxidizers under different pHs

**Figure 1:**
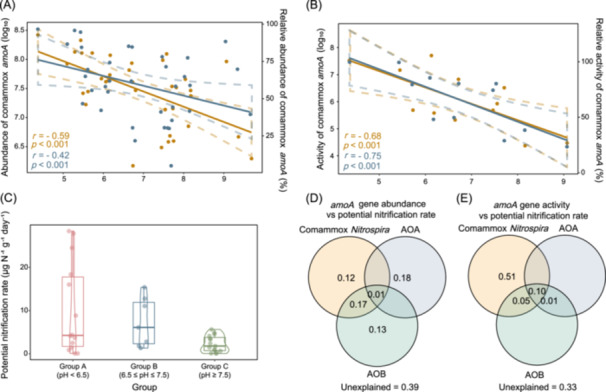
The relationship between the abundance and activity of comammox *Nitrospira* and pH. (A) The relationship between pH and the abundance and relative abundance of comammox *Nitrospira amoA;* 36 samples were used. The abundance of comammox *Nitrospira amoA* was expressed as copies per gram of dry soil and has been log‐transformed. The relative abundance of comammox *Nitrospira amoA* referred to the proportion of comammox *Nitrospira amoA* copies relative to the total copies of ammonia oxidizers *amoA* in each sample. Each data point represented the average of three technical replicates. Dashed line represents the 95% confidence interval (CI). (B) The relationship between pH and the activity and relative activity of comammox *Nitrospira amoA;* 12 samples were used (mixed three replicates in one). The activity of comammox *Nitrospira amoA* was expressed as copies per gram of dry soil and has been log‐transformed. The relative activity of comammox *Nitrospira amoA* referred to the proportion of comammox *Nitrospira amoA* copies relative to the total copies of ammonia oxidizers *amoA* in each sample. Each data point represented the average of three technical replicates. Dashed line represents the 95% CI. (C) The potential nitrification rate. Each data point represented the average of three technical replicates. (D) The effect of different ammonia oxidizers abundance on potential nitrification rate (calculated by variance partitioning analysis (VPA), Individual). The results for univariate, bivariate, and trivariate variables (Partition) can be observed in Table S6. (E) The effect of different ammonia oxidizers activity on potential nitrification rate (calculated by VPA analysis, Individual). The results for univariate, bivariate, and trivariate variables (Partition) can be observed in Table S7. AOA, ammonia‐oxidizing archaea; AOB, ammonia‐oxidizing bacteria.

Firstly, we collected soil samples with pH values ranging from 4.4 to 9.7 in China (Figures S1−S3 and Tables S1, S2) and quantified the abundance and activity of ammonia oxidizers’ *amoA* (Figure 1 and Figure S4). Results showed that comammox *Nitrospira* exhibited consistently high abundance and activity across all three pH groups analyzed (Figure S4). Comammox *Nitrospira* were dominant ammonia oxidizers in 63.8% of samples, followed by AOA (36.1%). Remarkably, the abundance and activity of comammox *Nitrospira amoA* displayed a distinct pH preference, with both abundance and activity peaking in Group A (samples with pH < 6.5) and demonstrating a significant negative correlation with pH (Figure 1A,B). Multiple statistical analyses were conducted with various environmental factors as the independent variables and the comammox *Nitrospira amoA* gene copy number as the dependent variable (Figures S5−S8 and Tables S3−S5). These analyses consistently confirmed that pH was the most influential factor affecting the abundance of comammox *Nitrospira*. In contrast to comammox *Nitrospira*, no significant pH preferences could be observed for other ammonia oxidizers (i.e., AOA and AOB) within the collected samples (Figure S4). We also determined the potential nitrification rate (Figure 1C) and investigated the contributions of different ammonia oxidizers to this process through variance partitioning analysis. Results showed that although the abundance of comammox *Nitrospira* explained only 28% of the variation in potential nitrification rate (Figure 1D and Table S6), its activity explained 70% (Figure 1E and Table S7), suggesting that comammox *Nitrospira* might dominate the ammonia oxidation in soil. Overall, in weakly acidic soil, comammox *Nitrospira* emerged as the most abundant and active ammonia oxidizer, with both its abundance and activity showing a strong negative correlation with pH.

### Ecological status of potential comammox *Nitrospira* amplicon sequence variants (ASVs)

**Figure 2:**
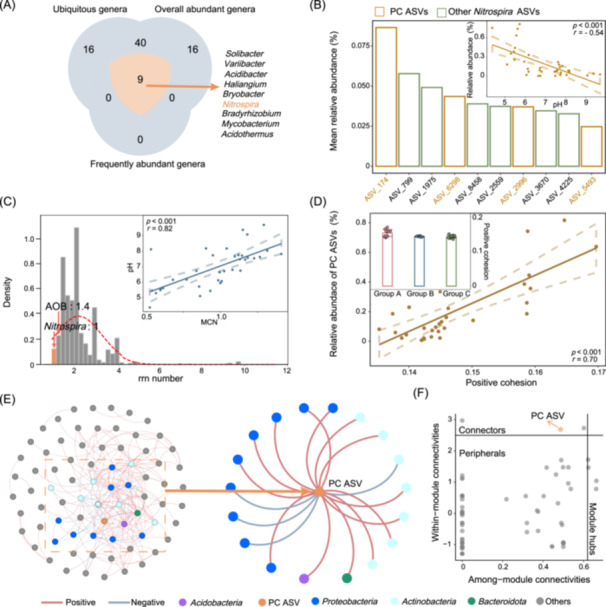
The ecological status for terrestrial potential comammox *Nitrospira*. (A) Screening for the core terrestrial genera (the detail of the criteria could be found in Methods). *Nitrospira* are highlighted in orange. (B) Identification of potential comammox amplicon sequence variants (PC ASVs) and their association with pH. The mean relative abundance represented the average value across all samples. The PC ASVs were highlighted in orange. The relationship between pH and mean copy number (MCN) is shown in the subfigure. (C) The distribution of rrn copy number and the relationship between MCN and pH. The mean rrn copy number for the *Nitrospira* and the detected AOB genera are shown. Details can be observed in Tables S8−S10. The relationship between pH and the relative abundance of PC ASVs is shown in the subfigure. (D) The relationship between the relative abundance of PC ASVs and positive cohesion. Changes in positive cohesion at different pH groups are shown in the subfigure. Dashed lines in (B)–(D) represent the 95% CI. (E) The network for bacteria. The blue lines indicate a negative correlation, and the red lines indicate a positive correlation. Gray nodes indicate no direct association with PC ASV. Different colors of the nodes indicate different taxonomies. PC ASV is highlighted in orange. PC ASV and ASVs directly connected to PC ASVs are shown in the subnetwork. (F) Zi−Pi analysis. The orange node indicates PC ASV. Black font identifies the node type.

To further reveal how pH influences comammox *Nitrospira* and its ecological status in the bacterial community, all the samples underwent high‐throughput 16S rRNA gene sequencing (V4 region). The rarefaction curve showed that the sequencing depth was adequate for the high‐throughput sequencing (Figure S9). The top 10 genera in different pH groups are shown in Figure S10. We combined three measures to screen for the core genera of the topsoil bacterial community, including ubiquitous genera (present in more than 80% of total samples), overall abundant genera (average relative abundance >0.1%), and frequently abundant genera (relative abundance above 80% in more than 50% of samples). Only 9 genera fulfilled all of these criteria, and *Nitrospira* was selected (Figure 2A). High‐throughput sequencing is an effective means to infer bacterial ecological status, but it was difficult to distinguish comammox *Nitrospira* from traditional *Nitrospira* using 16S rRNA gene sequencing. Thus, we combined phylogenetic analysis (Figure S11), random forest analysis, and correlation analysis (Figure S12), to identify four ASVs affiliated with Lineage II (i.e., ASV174, ASV6289, ASV2996, and ASV5493) as potential comammox ASVs (PC ASVs). Results showed that the sum of the relative abundance of these PC ASVs was highly associated with pH (*r* = −0.54, *p* < 0.05) (Figure 2B). The significant negative correlation with pH was observed only in the PC ASVs, while no such correlation was found in the groups of total *Nitrospira*, total *Nitrospira* excluding PC ASVs, or *Nitrospira* lineage II excluding PC ASVs (Figure S13). These results demonstrate that the PC ASVs exhibit a unique association with pH (Figure 2B).

To ascertain the adaptability of these PC ASVs under low pH conditions (i.e., as slow‐growing but efficient oligotrophs or fast‐growing copiotrophs), we evaluated their maximum growth rates. Given that the ribosomal RNA operon (rrn) copy number serves as a proxy for maximum growth rate and is a phylogenetically conserved trait [29, 30], the rrn copy number for each ASV was determined by matching the Ribosomal RNA Operon Copy Number Database (rrndb, version 5.6) to infer their respective maximum growth rates (Figure 2C). Results showed that all *Nitrospira* species encode a single 16S rRNA operon gene, which is lower than the average of 1.4 observed in AOA and AOB (Tables S8−S10). It suggested that comammox *Nitrospira* are slow‐growing bacterial species within the terrestrial bacterial community, further implying that low pH conditions may favor the selection of slow‐growing bacteria. To confirm this, we further calculated the mean copy number (MCN) [31], an abundance‐weighted rrn copy number, to examine how pH influenced the distribution of fast‐growing and slow‐growing taxa in terrestrial bacterial communities. Subsequently, a significant positive correlation could be observed between MCN and pH (*p* < 0.001, *r* = 0.82) (Figure 2C). Thus, low pH might be selected for bacteria characterized as slow‐growing but efficient oligotrophs, with comammox *Nitrospira* serving as a representative group.

To explore the broader ecological implications, we calculated the positive cohesion (indicating the degree of potential bacterial cooperation for each sample) and negative cohesion (indicating the degree of potential bacterial competition for each sample). The results demonstrated that potential bacterial cooperation might be promoted under acidic soil conditions (Figure 2D and Figure S14), as evidenced by a strong positive correlation between the relative abundance of PC ASVs and positive cohesion (Figure 2D), rather than negative cohesion (Figure S15). To ensure that this phenomenon was unique to PC ASVs rather than common to the *Nitrospira* whole genus, the relationships between the relative abundance of different *Nitrospira* groups (total *Nitrospira*, *Nitrospira* without PC ASVs, and *Nitrospira* lineage II excluding PC ASVs) and positive cohesion were analyzed (Figure S16). The results showed that only the relative abundance of PC ASVs was highly correlated with positive cohesion. These results implied that comammox *Nitrospira* might promote bacterial cooperation in low pH.

To validate these observations, we constructed a network analysis to visualize the relationships between comammox and other bacteria (Figure 2E). The combination of the inference of direct and indirect relationships with effective copula‐based transitivity (iDIRECT) [32], Goberna's method (Figure S17), and the link test for environmental filtering (LTEF) (Figure S18) confirmed that the links observed in the constructed network were due to real bacterial interactions rather than environmental filtering [25, 33]. Meanwhile, the Zi (within‐module connectivity) −Pi (among‐module connectivity) value was also calculated to determine the ecological status of each node at the ASV level (Figure 2F). Results showed that one PC ASV could be observed in the network and directly linked to 18 nodes (24% of the total number of nodes), and these nodes were affiliated with four phyla, including *Proteobacteria* and *Acidobacteria*, among others. Remarkably, all of these correlations were positive, implying potential cooperative interactions between PC ASV and these phyla (Figure 2E). Zi−Pi analysis indicated that this PC ASV acted as a key node (connectors) within the soil bacterial community (Figure 2F). Overall, these PC ASVs affiliated with Lineage II might be the key species selected by low pH, and may promote bacterial cooperation in weakly acidic soil.

### Metagenomic sequencing and composition of MAGs

**Figure 3:**
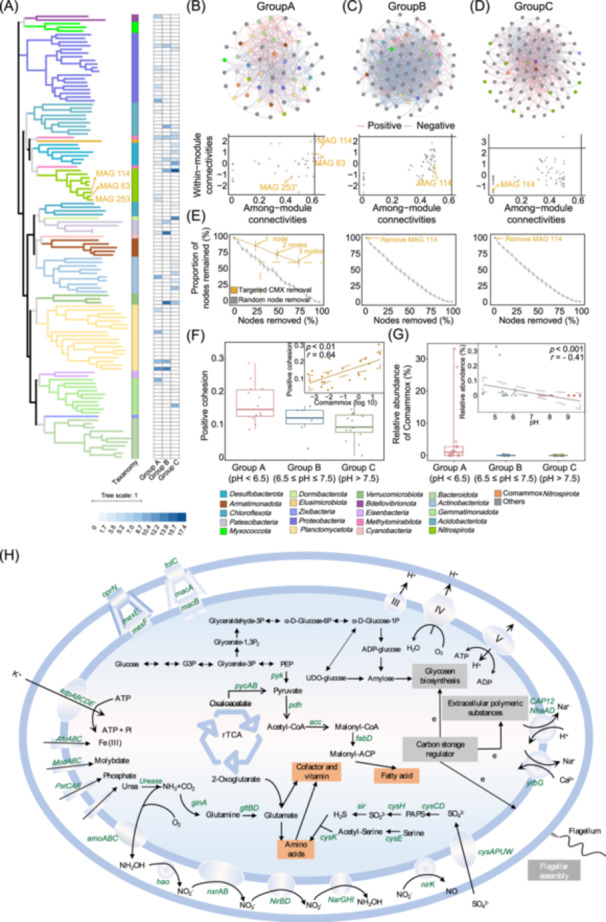
The metabolic potential for comammox *Nitrospira*. (A) Phylogenetic analysis of 121 metagenome assembled genomes (MAGs). Comammox *Nitrospira* MAGs are highlighted in orange (i.e., MAG 63, MAG 253, and MAG 114). The inner section indicated the taxonomy of each MAG. The outer section indicated the relative abundance of each MAG in each group. (B) The potential bacterial interaction and Zi−Pi analysis in Group A. Gray nodes in the network indicate that there is no direct connection with comammox *Nitrospira* MAGs, whereas nodes of other colors represent different phyla (directly linked with comammox *Nitrospira* MAGs), with orange specifically denoting comammox *Nitrospira* MAGs. The blue lines indicate a negative correlation, and the red lines indicate a positive correlation. (C) The potential bacterial interaction and Zi−Pi analysis in Group B. (D) The potential bacterial interaction and Zi−Pi analysis in Group C. (E) The random node removal and targeted node removal in the constructed networks. Gray lines indicated random node removal. The orange line represented targeted node removal, which included only comammox *Nitrospira* MAGs, with each node highlighted in orange font. (F) The positive cohesion in different pH groups. The positive cohesion is calculated by the relative abundance of MAGs. The relationship between positive cohesion (calculated by MAGs) and the relative abundance of comammox *Nitrospira* MAGs is shown in the subfigure. (G) The relative abundance of comammox *Nitrospira* MAGs in different pH groups. The relationship between pH and the relative abundance of comammox *Nitrospira* MAGs is shown in the subfigure. The relationship between pH and the relative abundance of commamox *Nitrospira* MAGs excluding outliers is shown in Figure S21. Dashed lines in (F) and (G) represent the 95% CI. (H) Cell metabolic diagram constructed from the soil comammox *Nitrospira* genome annotations. Only genes relevant for the carbon, nitrogen, energy metabolisms, and nutrient transport are shown. ADP, Adenosine Diphosphate; ATP, Adenosine Triphosphate; e, electron; EPS, extracellular polymeric substances; G3P, Glyceraldehyde‐3‐phosphate; PAPS, 3′‐Phosphoadenosine 5′‐phosphosulfate; PEP, phosphoenolpyruvate; Pi, Inorganic Phosphate; rTCA, reverse tricarboxylic acid cycle; III, Complex III; IV, Complex IV; V, Complex V.

Although the high‐throughput results provided insights into the presence of comammox *Nitrospira* in the community, more accurate methods are still required for confirmation. To further reveal the ecological status of comammox *Nitrospira*, we performed metagenomic sequencing on a total of these 36 samples. After co‐assembly and binning, we obtained 432 MAGs. Only high‐quality and middle‐quality MAGs (121 MAGs in total) were included in further analyses [34]. Based on the phylogenetic analysis using the Genome Taxonomy Database (GTDB, v214) [35], only bacterial MAGs were obtained, affiliated to 21 different phyla (Figure 3A). Of these, 9 *Nitrospira* MAGs were obtained. To further confirm whether these genomes were comammox *Nitrospira*, we downloaded 538 *Nitrospira* genomes and performed phylogenetic analyses of the 9 *Nitrospira* MAGs we obtained (Figure S19). Results showed that MAG 63, MAG 114, and MAG 253 were affiliated with comammox *Nitrospira* Clade A (Figure S19). Their *amoA* genes were consistent with the *amoA* genes of the current known comammox *Nitrospira* (Figure S20). The concurrent presence of *amoA*, *hao*, and *nxrA* genes in these 3 MAGs indicated that these MAGs encoded the complete nitrification pathway and were affiliated with comammox *Nitrospira*. Overall, we obtained 3 MAGs affiliated to comammox *Nitrospira* in our samples (Figure 3A).

To reveal the potential microbial interactions and the ecological status of comammox *Nitrospira*, we constructed the network and calculated the Zi−Pi value (Figure 3B−D). Results showed that comammox *Nitrospira* only acted as a key species in Group A (samples with pH < 6.5), and it is directly connected to 32 nodes, which is significantly higher than that in Group B (18 nodes) and Group C (5 nodes). We simulated the removal of the comammox *Nitrospira* nodes to explore their effect on the bacterial community (Figure 3E). The results showed that the absence of comammox *Nitrospira* node (only MAG 114) in Groups B and C had a minimal impact on the community, similar to the removal of a single random node. Remarkably, in Group A, the removal of comammox *Nitrospira* nodes led to the disappearance of 38% of the nodes in the molecular network, an impact equivalent to the effect of removing 30% of all nodes. This suggested that comammox *Nitrospira* may play a crucial role in maintaining community stability in weakly acidic soils through potential interactions with other bacteria.

To further confirm the importance of comammox *Nitrospira* for inter‐bacterial cooperation, we calculated positive cohesion and explored its relationship with comammox *Nitrospira* (Figure 3F). The results are consistent with that obtained by high‐throughput sequencing, that is, positive cohesion peaked in Group A and was positively associated with the relative abundance of comammox *Nitrospira* (Figure 3F). Meanwhile, the significant negative correlation between pH and comammox *Nitrospira* was also confirmed again in metagenomic sequencing (Figure 3G). Comammox *Nitrospira* were abundant at low pH, with a relative abundance that was more than 9.9 − 2139.5 times higher than that in the neutral (0.53%) and alkaline (0.0024%) soils, and the correlation remained even when outliers were excluded (Figure S21). In conclusion, the metagenomic results are consistent with those obtained by high‐throughput sequencing and quantitative real‐time PCR (qPCR), confirming that comammox *Nitrospira* favor low pH and might promote bacterial cooperation in weakly acidic soils.

To infer the metabolic potential of the obtained MAGs, the genes in each MAG were annotated by the Kyoto Encyclopedia of Genes and Genomes (KEGG) Orthology (KO). In the obtained bins, nitrogen metabolic pathways common in other comammox *Nitrospira* were observed (e.g., urea metabolic, complete nitrification) (Figure 3H). Remarkably, *NirBD* and *NarGHI*, which encode the functions of nitrate reductase and nitrite reductase, were present in the terrestrial comammox *Nitrospira* MAGs, suggesting that they had the potential for nitrate reduction assimilation. The rTCA cycle was the only complete carbon fixation pathway in the reconstructed bins, implying that comammox *Nitrospira* might use this process to fix CO_2_. For adaptation to acidic pH, the obtained comammox *Nitrospira* MAGs encoded the potassium transport system (*kdpABC*), which is involved in maintaining the reverse membrane potential in acidophiles through active influx of K^+^ [36]. The obtained MAGs also encode Na^+^/H^+^, K^+^/H^+^, and Na^+^/Ca^+^ antiporters, which are widely used acid resistance strategies to pump out protons [37]. Moreover, the obtained MAGs contained three sets of genes encoding F‐type ATPase, implying that comammox *Nitrospira* could export excess protons at acidic pH like other acid‐tolerant/acidophilic microbes. The complete gene sets encoding flagellar biosynthesis, EPS synthesis, and glycogen biosynthesis could be found in the reconstructed bins, which showed that they could form a biofilm to help tolerate low pH. Moreover, we also found 2 proton motive force (PMF) efflux pump (MexEF‐OprN and MacAB‐TolC) in the constructed comammox *Nitrospira* MAGs, whose running force is composed of the pH and electrochemical potential inside and outside the cytoplasmic membrane [38].

### Analysis of metabolic potential

**Figure 4:**
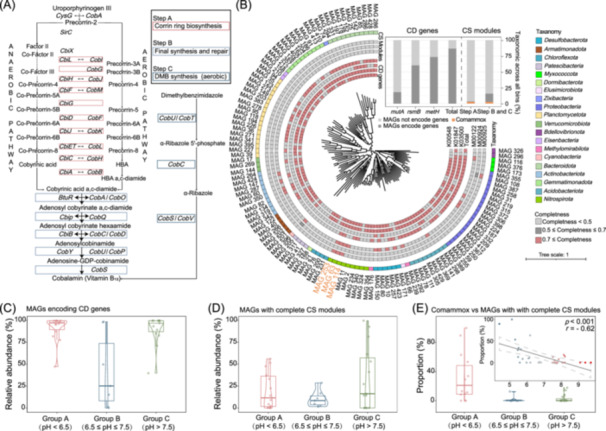
The biosynthesis and demand for cobalamin in soil. (A) The biosynthesis pathway of cobalamin in soil. Dashed lines indicate the absence of the gene, while solid lines indicate its presence. (B) Cobalamin‐dependent genes (CD genes) and cobalamin synthesis modules (CS modules) are encoded by each MAGs. Red indicates the presence of the CD gene and the completeness of the CS modules, while gray indicates their absence or incompleteness. The subfigure indicates the proportion of MAGs encoding *mutA*, *metH*, and *rsmB* (i.e., CD genes), and those containing genes for the various stages of the CS modules (Step A, Step B, and Step C). Total refers to the number of MAGs containing each of these three genes. Comammox *Nitrospira* are highlighted in orange. (C) The relative abundance of MAGs encoding CD genes under different pH. (D) The relative abundance of MAGs with complete CS modules. (E) The proportion of the relative abundance of comammox *Nitrospira* in each sample relative to the relative abundance of MAGs with complete CS modules. The relationship between pH and the proportion (the proportion of the relative abundance of comammox *Nitrospira* in each sample relative to the relative abundance of MAGs with complete CS modules) is shown in the subfigure. Dashed line represents the 95% CI.

To infer the metabolic potential of the obtained MAGs, we annotated the genes in each MAG using KO. For synthesis, we focused on the modules related to the biosynthesis of amino acids (25 modules), lipids and fatty acids synthesis (11 modules), and vitamins and cofactors (29 modules). Overall, the Comammox *Nitrospira* MAGs encoded 40 complete modules, which was 1.8 times higher than other MAGs (~22.9). Remarkably, all comammox *Nitrospira* MAGs encoded the complete cobalamin biosynthesis pathway, a rare function encoded by only the three comammox *Nitrospira* MAGs and one *Reyranella* MAG (Figure 4A,B). To reveal the supply and demand for cobalamin, we focused on 3 genes encoding cobalamin‐dependent enzymes as indicators of cobalamin dependence, including *mutA* (methylmalonyl‐CoA mutase), *rsmB* (ribosomal small subunit methyltransferase), and *metH* (methionine synthase). Results showed that more than 86.8% of the MAGs encoded at least one of these genes, suggesting that cobalamin dependence was very common in the soil bacterial community (Figure 4B). In contrast, only 4 MAGs harbored genes for the entire cobalamin synthesis pathway, and the other 15 MAGs encoded the final synthesis and repair (Step B) and Dimethylbenzimidazole synthesis (Step C) pathway.

We further revealed how cobalamin‐dependent MAGs (CD MAGs, MAGs encoding at least one cobalamin dependence gene) and cobalamin synthesis MAGs (CS MAGs) responded to pH. The results showed that both CD MAGs and CS MAGs peaked in acidic (~92.2%, ~20.0%) and alkaline (~90.9%, ~33.5%) soils (Figure 4C,D). To reveal how comammox *Nitrospira* MAGs contributed, we calculated the proportion of comammox *Nitrospira* to CS MAGs in different samples (Figure 4E). Results showed that comammox MAGs contributed the most in acidic soils (~31.6%), which was more than 15.0 times higher than that in neutral (2.1%) and alkaline (3.0%) soils. Meanwhile, a significant negative correlation was observed between the proportion of comammox *Nitrospira* to CS MAGs and pH (*p* < 0.001, *r* = −0.62). Thus, at the MAGs level with high and medium completeness, comammox *Nitrospira* were the dominant provider of cobalamin in weakly acidic soil. Considering the complexity of the soil bacterial community, although retaining high/medium quality MAGs was beneficial for the analysis of metabolic pathways, it also filtered out many contigs that cannot be assembled into high/medium quality MAGs. To ensure the reliability of the results, an analysis at the contigs level was performed. The results confirmed those obtained at the MAGs level (Figure S22). Overall, these results showed that comammox *Nitrospira* might be the key species in acidic soils, acting as one of the main cobalamin providers under low pH, based on the metagenomic analysis.

### Results of DNA‐SIP

**Figure 5:**
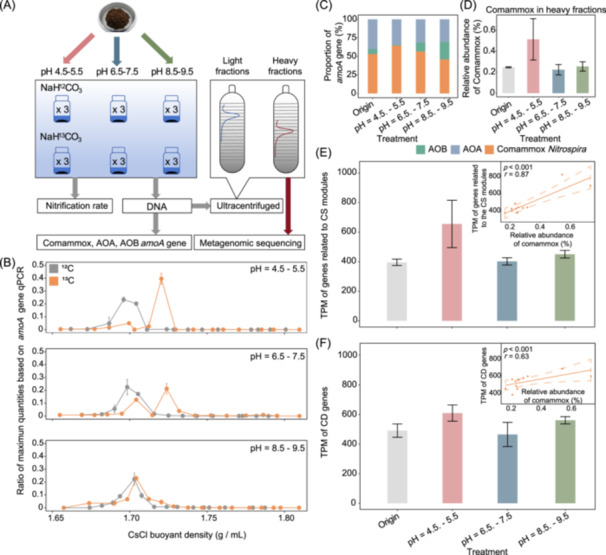
DNA stable‐isotope probing (DNA‐SIP) microcosms. (A) Pipeline for DNA‐SIP microcosms. (B) The “heavy” and “light” fractions under different pHs based on the quantitative real‐time PCR (qPCR) for comammox *Nitrospira amoA*. Line graphs represent the mean ± standard deviation (SD), with error bars indicating the SD. (C) The proportion of AOA, AOB, and comammox *Nitrospira amoA* in “heavy” fractions. Origin refers to the untreated soil. (D) The relative abundance of comammox *Nitrospira* (obtained by short reads data) in ‘heavy’ fractions. (E) Transcripts per million (TPM) of genes related to the biosynthesis of cobalamin. The relationship between the TPM of genes related to the biosynthesis of cobalamin and the relative abundance of comammox *Nitrospira* MAGs is shown in the subfigure. (F) TPM of cobalamin dependence (CD) genes. The relationship between the TPM of cobalamin dependence genes and the relative abundance of comammox *Nitrospira* MAGs is shown in the subfigure. Bar graphs in (D)–(F) represent the mean ± SD, with error bars indicating the SD. Dashed lines in (E) and (F) represent the 95% CI.

DNA‐SIP was used to confirm the results obtained from previous statistical analyses. Neutral soil (pH = 6.5−7.5) was selected and cultured under acidic (pH = 5), neutral (pH = 7), and alkaline (pH = 9) conditions, with NaH^13^CO_3_ (^13^C group) as marker and NaH^12^CO_3_ (^12^C group) as control (Figure 5A). After 56 days of cultivation, DNA was extracted and separated by density gradient centrifugation, allowing for the distinction of labeled organisms. qPCR was employed to quantify the comammox *Nitrospira*, AOA, and AOB *amoA* genes in the “heavy” and “light” fractions (Figure 5B, Figures S23−S27). First, the copy numbers of *amoA* genes across all fractions were aggregated. The results revealed a similarity between the ^13^C group and the ^12^C group, suggesting that the disparity in the *amoA* gene content across different fractions was driven by the assimilation of ^13^C rather than variations in the sum of *amoA* gene copy numbers (Figure S23). A detailed comparison of the distribution of comammox *Nitrospira amoA* among the fractions was then conducted. The results demonstrated that comammox *Nitrospira* are capable of assimilating ^13^CO_2_ under both acidic and neutral conditions. This was evident from a distinct single peak observed in the location of the heavier layers with higher buoyancy density (Figure 5B), mirroring the pattern observed in AOA *amoA* (Figure S26). In contrast, under alkaline conditions, AOB dominated the ammonia oxidation process (Figures S25, S27). Given the notable differences observed between the light and heavy fractions, the relative abundance of the *amoA* gene within the heavy layer was subsequently examined, followed by metagenomic sequencing. Results showed that comammox *Nitrospira* were dominant ammonia oxidizers under acidic conditions, with *amoA* gene accounting for 64.4% of total amoA genes (qPCR) and relative abundance reaching 0.5% in total species (metagenomic sequencing), 1.2–2 times higher than the original sample (Figure 5C,D).

To confirm how comammox *Nitrospira* contributed to cobalamin synthesis, the transcripts per million (TPM) of genes related to biosynthesis (biosynthesis genes) and cobalamin dependence (dependence genes) were analyzed (Figure 5D,E). The TPM values for both biosynthetic and dependence genes peaked in the acidic condition (655.3, 609.8), whereas they were minimal in the neutral condition (402.3, 465.3). Meanwhile, the relative abundance of comammox *Nitrospira* was positively associated with the TPM of biosynthesis genes (*p* < 0.001, *r* = 0.87) and dependence genes (*p* < 0.05, *r* = 0.63) (Figure 5F). Overall, the DNA‐SIP results confirmed that comammox *Nitrospira* are dominant ammonia oxidizers under acidic conditions and may contribute to the biosynthesis of cobalamin in acidic soil.

Detailed DNA‐SIP results for AOA and AOB *amoA,* and the phylogenetic analysis of *amoA* are available in the Supplementary Information.

## DISCUSSION

Based on the survey of 36 soil samples with pH values ranging from 4.4 to 9.7, our study provides strong evidence that comammox *Nitrospira* are dominant ammonia oxidizers and key species under low pH conditions. Additionally, genomic evidence (at both MAGs and contig levels) suggests that comammox *Nitrospira* may cooperate with other bacteria under low pH, as it has been identified as one of the primary suppliers of cobalamin in weakly acidic soils.

Weakly acidic conditions promoted the growth and competitive advantage of comammox *Nitrospira*. Utilizing various sequencing technologies and statistical approaches, we confirmed that comammox *Nitrospira* are the most abundant and active ammonia oxidizers in weakly acidic soils, as evidenced by both in situ and microcosm tests. This result is also consistent with the speculations of Zhu et al. [18]. Environmental selection may make the greatest contribution to the dominance of comammox *Nitrospira*. (1) Low pH levels can potentially constrain substrate availability for ammonia oxidizers by affecting the dissociation equilibrium of ammonia [39]. According to estimates, the concentration of free ammonia in weakly acidic soil is approximately 0.006 μM NH_3_ (from microcosm tests) (Table S11). These concentrations were found to be lower than the semi‐saturation constant (*Ks*) for the representative strain (63 nM NH_3_) of comammox *Nitrospira* (*N. inopinata*) and significantly lower than the *Ks* for AOA and AOB [17]. Thus, lower free ammonia concentrations in weakly acidic soils might promote the ecological success of comammox *Nitrospira*. (2) Comammox *Nitrospira* may have unique metabolic pathways and acid tolerance genetics. According to the metabolic potential of the obtained MAGs, comammox *Nitrospira* encoded Na^+^/H^+^, K^+^/H^+^, and Na^+^/Ca^+^ antiporters, F‐type ATPase [20], PMF efflux pump [40], and genes related to biofilm formation to resist the low pH [41]. Moreover, comammox *Nitrospira* affiliated with Clade A may possess acidophilic or acid‐tolerant genotypes, enabling adaptation to acidic conditions. As shown in microcosm tests, the active comammox *Nitrospira* (“heavy” fractions) obtained in weakly acidic soil were evolutionarily distinct from those obtained in neutral or high pH soils (Figure S28). Phylogenetic analysis of known comammox *Nitrospira amoA* sequences showed that only comammox *Nitrospira* Clade A was observed in soils with pH lower than 5.5 and was evolutionarily distinct from other sequences (Figure S29). Although it remains unclear whether there are genotypes of comammox *Nitrospira* with specialized acidophilic attributes like those of AOA [8], our results led us to hypothesize it might be affiliated with comammox *Nitrospira* Clade A. That comammox *Nitrospira* Clade A enriched from acidic tea plantation soils [42], acidic mine lake [20], and an acidic reactor [19] could all oxidize ammonia under low pH further validate our results. Indeed, it is necessary to furnish direct evidence for the existence of acidophilic or acid‐tolerant genotypes by enriching and isolating comammox *Nitrospira* from acidic soils. (3) Low pH might favor slow‐growing bacteria. Distinguishing between fast and slow growers offers a robust trait‐based depiction of bacterial community structure [30, 31]. A significantly negative correlation was observed between pH and MCN across the surveyed pH range suggesting that the weakly acidic conditions might be conducive to slow‐growing bacteria (Figure 2). Both the rrn copy number for comammox *Nitrospira* and the significant correlation between PC ASVs and MCN suggested that comammox *Nitrospira* were one of the selected slow‐growing bacteria (Figure 2). This phenomenon can be attributed to the reduction in growth and biosynthesis during stressful conditions, such as low pH, where cellular resources are primarily directed toward stress tolerance and resource acquisition [24]. As a result, comammox *Nitrospira*, characterized by a lower maximum growth rate but higher yield, exhibits a competitive advantage over resource‐expending, fast‐growing competitors in weakly acidic soils [43]. However, the 16S rRNA copy number only reflects microbial growth potential, not actual growth rates. Growth rates are influenced by a combination of factors, including metabolic efficiency, environmental conditions, and resource availability. Therefore, further investigation is needed to confirm these ecological implications and evaluate the broader significance of this finding. This preference for traits focused on stress tolerance and resource acquisition enhances the fitness of comammox *Nitrospira* in weakly acidic soil.

Moreover, our results suggested that comammox *Nitrospira* might act as a key species in the soil bacterial community by sharing cobalamin as a public good. As a shared vitamin, cobalamin could affect microbial growth at low external concentrations, even at the picomolar level [44] (Figure 4). An imbalance between cobalamin supply and demand in the soil bacterial community can be observed, as only the comammox *Nitrospira* MAGs and *Reyranella* MAG encoded the complete synthesis pathway, yet more than 86.8% of the bacterial community requires it (Figure 4). As with the extensive dependence of bacteria on cobalamin in soil [45], extensive dependence of bacteria on cobalamin has been observed in other systems and environments, including marine [46, 47], wastewater treatment plants (WWTPs) [48], leaf‐associated bacteria [49], and even composting [25], where more than 80% of the microbes depend on cobalamin for growth but cannot synthesize it [50]. This imbalance can be supported by the Black Queen Hypothesis (BQH) [51, 52], which states that microbes can live more efficiently and benefit from other microbes by paring down burdensome genes. Soil is a moderately stable environment where low pH, apart from selecting specific species, may drive cooperative evolution among species. Indeed, cobalamin synthesis is a high metabolic burden for microbes [45], due to its complex biosynthesis (more than 30 enzymatic steps). The strong associations between the relative abundance of comammox *Nitrospira* and positive cohesion and the cobalamin synthesis potential implied that comammox *Nitrospira* might cooperate with other bacteria by sharing cobalamin (Figure 5). The intense cooperation in weakly acidic soil could also be supported by theoretical predictions such as SGH [53], which was originally proposed to describe the interactions between plants [26]. The abiotic stress highlighted in this hypothesis was consistent with the low pH stress in weakly acidic soil [26]. Meanwhile, sharing cobalamin might also be beneficial to ammonia oxidizers, as it might alleviate the stress resulting from the buildup of nitrite and NO within cells via over‐synthesis of NO_2_‐cobalamin [54]. Although this phenomenon has been found in AOA, it may also serve as a detoxification strategy for comammox *Nitrospira*. Further research is needed to confirm its relevance in comammox *Nitrospira*. Moreover, it might also promote the formation of a biofilm, which in turn could promote the growth of comammox *Nitrospira* [55]. Although genomic evidence (from both contigs and MAGs) suggested that comammox *Nitrospira* might be the dominant provider of cobalamin in weakly acidic soils. Due to the complexity and diversity of the soil bacterial community, some rare species that could not be assembled into high/medium‐quality MAGs or long contigs might also contribute to the biosynthesis of cobalamin in weakly acidic soils. Future research could address this by increasing sequencing depth or utilizing single‐cell sequencing to differentiate the contributions of these rare species to cobalamin production in acidic soils.

Based on the analysis of 36 collected samples, it showed that comammox *Nitrospira* were the most abundant ammonia oxidizers, dominating over 63.9% of the samples (Figures 1, S4). This contrasts with previous studies, which often report AOA as the dominant ammonia oxidizer in unmanaged soils [22, 56]. This discrepancy might be attributed to the broad pH adaptation of comammox *Nitrospira*. Although both the in situ and microcosm tests showed that high pH inhibited the abundance and activity of comammox *Nitrospira*, the growth of comammox *Nitrospira* under alkaline conditions was also detected. No significant difference was observed between the comammox *Nitrsopira amoA* obtained from neutral or alkaline conditions (Figures S28, S29). Together, these results suggested that several comammox *Nitrospira* could tolerate alkaline conditions. The existence of these comammox *Nitrospira* might explain why the abundance of comammox *Nitrospira* increased in several alkaline forest and farmland sites [11, 22]. The wide soil pH adaptation and low pH preference of comammox *Nitrospira* suggested that it is possible to predict its biogeographic distribution from global soil pH data. A 5%−7% increase in the relative abundance of comammox *Nitrospira* was observed per 1‐unit decrease in pH. Currently, acidic soils constitute nearly 30% of the global soil area, and this proportion continues to rise [57]. Thus, comammox *Nitrospira* is expected to become increasingly important in global soils. The global distribution of comammox *Nitrospira*, coupled with their adaptation to low pH and low ammonia concentrations, underscores their critical role in soil nitrogen cycling. Furthermore, promoting comammox *Nitrospira‐*dominated communities could enhance nitrogen use efficiency and reduce nitrous oxide (N₂O) emissions [58], offering a practical avenue for sustainable soil management. Finally, although we employed various methods (phylogenetic analysis, random forest model, correlation analysis) to screen comammox ASVs from high‐throughput sequencing of the 16S V4 region and confirmed that they exhibit unique characteristics compared to other *Nitrospira* ASVs, further verification is still required. Given the challenges in recovering complete 16S sequences from MAGs obtained through short‐read sequencing, long‐read sequencing may provide an effective solution.

Overall, our results highlighted the importance of comammox *Nitrospira* in weakly acidic soils and revealed the mechanisms of its dominance in weakly acidic soils. Indeed, further pure culture‐based studies are needed to verify the status of comammox *Nitrospira* under acidic conditions and its association with other bacteria.

## CONCLUSION

Various genomic analyses and DNA‐SIP microcosm tests provided evidence that comammox *Nitrospira* were the K‐strategy species (i.e., slow growth and stress‐tolerant characteristics) in weakly acidic soil and had the potential to cooperate with other species. Considering its high abundance and occurrence frequency, comammox *Nitrospira* are also the core species in weakly acidic soil. Moreover, comammox *Nitrospira* are the unique species encoding the complete metabolic pathway for the biosynthesis of cobalamin, which can address the imbalance between supply and demand for cobalamin in the soil microbial community. Thus, we suggested that comammox *Nitrospira* might dominate the weakly acidic soil via its K‐strategy and its unique metabolic potential, which might form intense cooperation with other bacteria.

## METHODS

### Location and sample collection

To reveal how pH influenced comammox *Nitrospira*, soil samples with pH values ranging from 4.4 to 9.7 were collected from various locations in China (Table S1). Soil samples were collected from three major land use types (i.e., forest (Figure S1), grassland (Figure S2), and cropland (Figure S3)). To eliminate heterogeneity, each sample was a combination of three biological replicates spaced greater than 20 m apart. A tubular soil sampler (5 cm diameter, 60 cm length) was used to collect the samples. Overall, 36 samples (12 soil samples, 3 replicates) were collected for further study. The sample was divided into three groups based on their pH value, including Group A (pH < 6.5), Group B (6.5 ≤ pH ≤ 7.5), and Group C (pH > 7.5). The pH and nutrient contents of the sample can be observed in Table S2.

### DNA extraction, RNA extraction, and qPCR

A total of 36 samples were extracted with the DNeasy PowerSoil Kit (12888‐50, Qiagen), according to the manufacturer's guidelines. Before sequencing, DNA concentration, and purity were tested by Nanodrop One (Thermo Fisher Scientific, USA) and Qubit 2.0 (Thermo Fisher Scientific), respectively.

A total of 12 (mixed three replicates in one) samples were selected for RNA extraction using the RNeasy PowerSoil Kit (12866‐25, Qiagen). RNA concentration was quantified using the Qubit instrument (Thermo Fisher Scientific). To eliminate residual DNA and facilitate the reverse transcription of RNA into cDNA, the PrimeScript RT Master Mix Kit (RR047A, TaKaRa) was employed.

Both DNA and cDNA were detected, which could reflect the *amoA* gene abundance and activity for AOA, AOB, and comammox *Nitrospira*. The specific primers and annealing temperatures are listed in Table S3. qPCR amplification was conducted using the iCycler iQ5 instrument (BioRad), with the pre‐mixing system and program referenced from Hu et al. [59]. Plasmid quantification of AOA, AOB, and comammox *Nitrospira amoA* was performed using plasmids carrying the target genes, which were cloned from PCR‐amplified samples using the pMD19‐T vector (6013, TaKaRa). The amplification efficiency of the plasmid standard curve ranged from 88% to 95%, with an *R* ^2^ value of >0.99. The relative abundance (activity) of comammox *Nitrospira amoA* was calculated as: abundance (activity) of comammox *Nitrospira amoA*/sum of abundance (activity) of AOA, AOB and comammox *Nitrospira amoA*.

### Determination of physicochemical factors and potential nitrification rate

The soil temperature was measured in situ using a digital thermometer (MR‐10H, China). ${\mathrm{NH}}_{4}^{+}$‐N, ${\mathrm{NO}}_{2}^{-}$‐N, and ${\mathrm{NO}}_{3}^{-}$‐N were determined by spectrophotometry [27]. Soil pH was measured using a pH meter (Mettler‐Toledo) under a 1:2.5 soil‐to‐water ratio condition. Moisture content (MC) was determined by oven drying at 105°C. The carbon/nitrogen ratio was analyzed using an elemental analyzer (Vario Micro, Elementar Analysensysteme GmbH) [59]. We incubated a mixture of 5 g of soil and 50 mL of medium in the dark at 25°C with shaking (120 rpm) to test the potential nitrification rate. The medium was prepared as described by Daims et al. [13], combining 26.7 mg/L NH_4_Cl, 50 mg/L KH_2_PO_4_, 75 mg/L KCl, 50 mg/L MgSO_4_ × 7 H_2_O, 584 mg/L NaCl, 1000 mg/L CaCO_3_, and 1 mL of trace elements. Daily measurements of nitrite and nitrate concentrations were conducted. The nitrification rate was calculated based on the linear change in the sum of nitrite and nitrate concentrations over time [60]. The relationship between environmental factors and the abundance of comammox *Nitrospira* was confirmed through various methods.

### High‐throughput sequencing

We used primer pair 515F'/806R' (515F' GTGCCAGCMGCCGCGGTAA, 806 R' GGACTACHVGGGTWTCTAAT) to amplify the 16S rRNA V4 region as suggested by EMP [61]. After generating the sequencing library with NEBNext® Ultra™ DNA Library Prep Kit for Illumina (E7103, New England Biolabs), the library was sequenced by Illumina MiSeq (MAGIGENE) to generate 250 bp paired‐end reads. The PCR reaction system is described in Table S3. All 36 samples were analyzed following a consistent process. Each sample is the average of three technical replicates. In detail, Divisive Amplicon Denoising Algorithm 2 (DADA2, v1.8) was utilized to identify ASVs through clustering or de‐duplication (100% similarity threshold) [62]. The downstream data analysis was conducted using QIIME2 (version 2020.11.0). A total of 105,308 ASVs were obtained, each of which was matched to SILVA 138.1 for its taxonomy. After being resampled, a standardized ASV table was eventually obtained with 14,527 readings per sample. The profile of high‐throughput sequencing can be observed in Figures S9, S10.

Three criteria were used to screen the core terrestrial genera, including overall genus abundance, genus ubiquity, and frequently abundant genera [63]. An overall abundant genus should be among the top 0.1% genera with the highest mean relative abundance. A ubiquitous genus should be detected in over 80% of samples. A frequently abundant genus should rank in the top 80% in more than 50% of the samples. Since the 16S V4 region cannot distinguish between comammox *Nitrospira* and canonical *Nitrospira*, we combined three methods to identify comammox ASVs: (1) using phylogenetic analysis to find ASVs belonging to *Nitrospira* lineage II (Figure S11), (2) constructing a random forest model to identify *Nitrospira* lineage II ASVs that were highly correlated with comammox *Nitrospira amoA* copies (obtained by qPCR) (Figure S12), (3) testing different ASV group to reveal their unique characteristics (Figures S13−S16). Finally, these selected ASVs were referred to as PC ASVs.

### Metagenomic sequencing and data analysis

Sequencing libraries for the 36 DNA samples were constructed using NEBNext® Ultra™ DNA Library Prep Kit for Illumina (E7103, New England Biolabs). All DNA samples were sequenced on the Illumina NovaSeq. 6000 platform (MAGIGENE). On average, 1.24 × 10^10^ bases were obtained per sample (*n* = 36). Trimmomatic (v.0.36) was used to filter sequences (mass fractions < 20 base pairs and lengths < 50) [64] and 1.0 × 10^10^ clean data were obtained (Q20 = 100%, Q30 = 99.7%). We used MEGAHIT (v1.0.6, default parameter) to co‐assemble all samples to obtain contigs [65] and MetaBAT2 (v2.17) (sensitive parameter) to obtain MAGs [66] (including contig lengthen over 2000). MAG quality was assessed by CheckM (v2), whose quality standards were set according to the Genomic Standards Consortium (GSC). Only high‐quality MAGs (completeness >90%, contamination <5%) and medium‐quality MAGs (completeness ≥50%, contamination <5%) were retained for further study [34]. The relative abundance of MAGs was assessed based on the average coverage proportion of each MAG within a metagenomic sequencing sample. Genome Taxonomy Database Toolkit (GTDB‐Tk; v 2.3.0) was used to extract 120 ubiquitous single‐copy proteins and classify the taxonomic via GTDB (v214) [35]. The phylogenetic analysis was conducted via FastTree (v2.1) and visualized in iTOL v3.

### Molecular ecological network analysis and cohesion analysis

As the network reliability degraded significantly for ASVs containing >50% zeroes, ASVs that were present in a minimum of half of the total samples were retained for further analysis to bolster the robustness of the network [67]. The network was established based on Pearson correlations derived from log‐transformed abundances of ASVs. To minimize uncertainty in the construction of the network, an RMT (random matrix theory)‐based approach was used to identify the correlation cut‐off threshold [33]. As both environment filtering and microbial interaction could cause the correlations between taxa, we employed iDIRECT [32], a method specifically designed for network analysis, to distinguish between direct (microbial interactions) and indirect associations within networks. The iDIRECT was conducted on the molecular ecological network analysis pipeline (MENAP) interface (http://ieg4.rccc.ou.edu/MENA/). Moreover, to confirm whether the links observed in the network were driven by microbial interactions, Goberna's method and the LTEF were conducted [25, 33]. The details of Goberna's method and LTEF can be observed in Figures S17, S18. Overall, the used network has effectively minimized the indirect correlations caused by environmental filtering, thereby it could reflect potential microbial interactions more accurately. The topological indices were calculated through the MENAP interface (http://ieg4.rccc.ou.edu/MENA/). The complete network was visualized using Gephi (v0.10.1). The Zi−Pi method was used to confirm the key species in the network, which have high impacts on the structure and functioning of ecosystems, the thresholds following previously described [33]. Only ASVs with Zi ≥ 2.5 or Pi ≥ 0.62 were recognized as key ASVs.

We calculated the cohesion index to reveal “potential bacterial interactions” by similarities in the niches of each taxon [26]. The calculation method was as previously described. Briefly, the null model‐corrected correlations were determined by comparing the differences between pairwise correlations derived from those derived from multiple iterations of the null model and relative abundance. The recommended null model (i.e., “taxa shuffle”) was employed. The positive and negative cohesion of each sample were the respective sums of the product of the average of positive and negative correlations and the relative abundance for each species. The sum of cohesion is the sum of positive cohesion and the absolute values of negative cohesion. Since positive cohesion is a weighting of positive correlation and relative abundance, it can characterize potential bacterial cooperation. The sum of cohesion could reflect the degree of potential bacterial interactions, as it characterizes the total potential interactions [26, 33]. We randomly removed nodes and specifically targeted the removal of comammox *Nitrospira* nodes to reveal the importance of comammox *Nitrospira* in the bacterial community under different pH conditions. The abundance‐weighted mean interaction strength (wMIS) was calculated to assess the impact of species removal on the remaining community. After removing the chosen nodes, if wMIS ≤0, the species was deemed extinct or isolated and subsequently removed from the network, and the proportion of remaining nodes was calculated.

$${\mathrm{wMIS}}_{i}=\frac{\sum_{i\neq j}b_{j}s_{\mathrm{ij}}}{\sum_{j\neq i}b_{j}}$$

*b*_*j*_ represents the relative abundance of species *j*. *s*_*ij*_ denotes the interaction strength between species *i* and *j* (measured by *r* value).

### RNA operon copy number analysis and calculation of mean copy number

As the bacterium's maximum growth rate is proportional to its ribosomal RNA operon (rrn) copy number, we aligned all ASVs with rrndb (Ribosomal RNA Operon Copy Number Database) to obtain their rrn copy numbers. If the ASV is not classified at the species or genus level, we assign the rrn copy number based on its family‐level classification. To reveal the average growth rate in each sample, we calculated the MCN as described by Abreu et al. [31]. Briefly, the rrn copy number of each ASV was weighted by its relative abundance. The MCN for each sample represents the average of three replicates.

### DNA‐SIP microcosm design

Previous national survey shows the behavior of comammox *Nitrospira* in different pH soils. DNA‐SIP cultivation was used to reveal the effect of pH changes on the abundance and activity of comammox *Nitrospira*. To prevent the community from collapsing under excessive pH adjustment, neutral soils were selected and their pH values were adjusted to acidic and alkaline, respectively. Neutral soil samples were collected from Zhejiang Province (28°57'25.39″N, 119°38'45.78″E). The pH of the collected soil was 6.7. The soil samples were homogenized and mixed evenly through a 2.0 mm sieve. We constructed triplicate microcosms cultivation systems in 250 mL blue‐capped bottles with each containing 20 g of fresh soil. The soil was incubated at 25°C for 56 days in the dark [68]. We set up three groups, including weakly acid (pH 4.5−5.5), neutral (pH 6.5−7.5), and weakly alkaline (pH 8.5−9.5). Incubation of the soil microcosm was performed with ^13^C‐labeled and ^12^C‐labeled groups, with additions of 0.5 g/kg NaH^13^CO_3_ and NaH^12^CO_3_, respectively. In total, 18 microcosms were set up. 70 μg NH_4_Cl‐N were added into all microcosms as the nitrogen source. The treatment was renewed once a week, including adding 70 μg NH_4_Cl‐N g dry weight soil and 0.5 g/kg NaH^13^CO_3_ and Phosphate Buffer solution. Soil moisture content was maintained at 60% of its maximum water‐holding capacity [68]. Thus, the microcosms received a total of 560 μg NH_4_Cl‐N g dry‐weight gram soil over an incubation period of 8 weeks. After 56 days of cultivation, destructive sampling was performed for each treatment, and all the samples were divided into two parts for metagenomic sequencing and detection of nitrification rate.

### Density gradient centrifugation

DNA extraction, concentration, and purity assessment were as previously described. SIP fractionation was performed as described previously [69]. Briefly, we mixed 2.0 μg of DNA with CsCl stock solution and confirmed the refractive index reaching 1.4029. Afterward, the mixed CsCl solution was ultracentrifuged at 177,000 *g* for 48 h at 20°C. DNA fractionation was performed by displacing the gradient medium with sterile water from the top of the ultracentrifuge tube using a constant current pump (Longer Pump, LSP01‐1A). 16 DNA gradient fractions were obtained, and we used an AR200 digital hand‐held refractometer (Reichert, Inc.) to detect the refractive index for each fraction. The fractionated DNA was purified and dissolved in 30 μL TE buffer. We used qPCR to test the “light” and “heavy” fractions. The qPCR systems are described in Table S2. Due to the relatively small amount of purified DNA for each fraction, we combined DNA from “heavy” fractions of the same sample. Finally, 9 “heavy” fractions (3 groups labeled by ^13^C, 3 repeats) were sequenced using metagenomic sequencing. The sequencing details were as previously described. Kraken2 (v2.1.3) was used with short reads to reveal the relative abundance of comammox *Nitrospira* [70].

Detailed procedures for sample collection, sequencing protocol, data processing techniques for sequencing data, and bioinformatic and statistical analysis approaches are available in the Supplementary Information.

## AUTHOR CONTRIBUTIONS

**Yuxiang Zhao**: Conceptualization; investigation; validation; writing—original draft; writing—review and editing; formal analysis; visualization. **Jiajie Hu**: Conceptualization; methodology; writing—review and editing. **Jiaqi Wang**: Conceptualization; methodology; writing—original draft. **Xiangwu Yao**: Conceptualization; writing—original draft; methodology; data curation. **Tong Zhang**: Conceptualization; writing—original draft; writing—review and editing; methodology; software. **Baolan Hu**: Conceptualization; investigation; funding acquisition; writing—original draft; writing—review and editing; methodology; formal analysis; supervision; resources; project administration; visualization.

## CONFLICT OF INTEREST STATEMENT

The authors declare no conflicts of interest.

## ETHICS STATEMENT

No animals or humans were involved in this study.

## Supporting information


**Figure S1:** Sampling sites of forest soils.
**Figure S2:** Sampling sites of grassland soils.
**Figure S3:** Sampling sites of cropland soils.
**Figure S4:** The abundance and activity of ammonia oxidizers.
**Figure S5:** Random forest analysis.
**Figure S6:** Mantel test.
**Figure S7:** Hierarchical partitioning analysis.
**Figure S8:** Multiple linear regression between mean copy number (MCN) and environmental factors.
**Figure S9:** Rarefaction curves.
**Figure S10:** The overall community structure of the soil bacteria with the 10 most abundant taxa at the genus level.
**Figure S11:** Phylogenetic analysis of the *Nitrospira* Lineage II amplicon sequence variants (ASVs).
**Figure S12:** The screening for potential comammox *Nitrospira* ASVs (PC ASVs).
**Figure S13:** The relationship between total *Nitrospira* and *Nitrospira* excluding PC ASVs and pH.
**Figure S14:** The relationship between positive cohesion, negative cohesion, and the sum of cohesion and pH.
**Figure S15:** The relationship between PC ASVs and negative cohesion and the sum of cohesion.
**Figure S16:** The relationship between positive cohesion, negative cohesion, and the sum of cohesion and *Nitrospira*.
**Figure S17:** Quantification of the relative contribution of community assembly processes to molecular ecological network analysis (MENA) links.
**Figure S18:** Link Test for Environmental filtering (LTEF) for disentangling the contributions of environmental filtering to the observed network links.
**Figure S19:** The phylogenetic tree of all known *Nitrospira*.
**Figure S20:** The phylogenetic tree of *amoA* gene.
**Figure S21:** Relationship between pH and the relative abundance of comammox *Nitrospira* MAGs excluding outliers.
**Figure S22:** Abundance of cobalamin synthesis genes in contigs level.
**Figure S23:** The comammox *Nitropira amoA* gene copy number.
**Figure S24:** Nitrification rate in different groups.
**Figure S25:** CsCl buoyant density for AOB.
**Figure S26:** CsCl buoyant density for AOA.
**Figure S27:** *amoA* gene copy number for AOA and AOB.
**Figure S28:** Phylogenetic tree of active ammonia oxidizers.
**Figure S29:** Phylogeny of soil comammox *amoA* genes and their corresponding pH.

**Table S1:** Sample site information.
**Table S2:** Physicochemical factors for each sample.
**Table S3:** The qPCR primers used in this study.
**Table S4:** Linear regression analysis.
**Table S5:** VPA analysis result for the 4‐factor combination of pH, NO_2_^‐^/NO_3_^‐^, Sulfur, and Altitude to the abundance of comammox *Nitrospira*.
**Table S6:** VPA analysis result for the 3‐factor combination of the abundance of comammox *Nitrospira amoA*, AOA *amoA*, and AOB *amoA* to potential nitrification rate.
**Table S7:** VPA analysis result for the 3‐factor combination of the relative activity of comammox *Nitrospira amoA*, AOA *amoA*, and AOB *amoA* to potential nitrification rate.
**Table S8:** rrn copy number for *Nitrospira*.
**Table S9:** rrn copy number for AOB.
**Table S10:** rrn copy number for AOA.
**Table S11:** The predicted free ammonia concentration.

## Data Availability

The raw data for 16S rRNA gene high‐throughput sequencing and metagenomic sequencing have been deposited in the National Center for Biotechnology Information (NCBI) Sequence Read Archive (SRA) database under accession number PRJNA877822 (https://www.ncbi.nlm.nih.gov/bioproject/PRJNA877822) and PRJNA897831 (https://www.ncbi.nlm.nih.gov/bioproject/PRJNA897831). The data and scripts for analysis are saved in GitHub https://github.com/Yuxiang-Zhao/Comammox_pH. Supplementary materials (results, methods, figures, tables, graphical abstract, slides, videos, Chinese translated version, and update materials) may be found in the online DOI or iMeta Science http://www.imeta.science/.
