# Supplementary material for: Comammox Nitrospira act as key bacteria in weakly acidic soil via potential cobalamin sharing
Source: Imeta. 2025 Feb 4;4(1):e271. doi: 10.1002/imt2.271 (PMC11865330; doi:10.1002/imt2.271)
Supplement: Supplementary file 1 — Figure S1: Sampling sites of forest soils. Figure S2: Sampling sites of grassland soils. Figure S3: Sampling sites of cropland soils. Figure S4: The abundance and activity of ammonia oxidizers. Figure S5: Random forest analysis. Figure S6: Mantel test. Figure S7: Hierarchical partitioning analysis. Figure S8: Multiple linear regression between mean copy number (MCN) and environmental factors. Figure S9: Rarefaction curves. Figure S10: The overall community structure of the soil bacteria with the 10 most abundant taxa at the genus level. Figure S11: Phylogenetic analysis of the Nitrospira Lineage II amplicon sequence variants (ASVs). Figure S12: The screening for potential comammox Nitrospira ASVs (PC ASVs). Figure S13: The relationship between total Nitrospira and Nitrospira excluding PC ASVs and pH. Figure S14: The relationship between positive cohesion, negative cohesion, and the sum of cohesion and pH. Figure S15: The relationship between PC ASVs and negative cohesion and the sum of cohesion. Figure S16: The relationship between positive cohesion, negative cohesion, and the sum of cohesion and Nitrospira. Figure S17: Quantification of the relative contribution of community assembly processes to molecular ecological network analysis (MENA) links. Figure S18: Link Test for Environmental filtering (LTEF) for disentangling the contributions of environmental filtering to the observed network links. Figure S19: The phylogenetic tree of all known Nitrospira. Figure S20: The phylogenetic tree of amoA gene. Figure S21: Relationship between pH and the relative abundance of comammox Nitrospira MAGs excluding outliers. Figure S22: Abundance of cobalamin synthesis genes in contigs level. Figure S23: The comammox Nitropira amoA gene copy number. Figure S24: Nitrification rate in different groups. Figure S25: CsCl buoyant density for AOB. Figure S26: CsCl buoyant density for AOA. Figure S27: amoA gene copy number for AOA and AOB. Figure S28: Phylogenetic tree of active ammonia [file IMT2-4-e271-s002.docx]

**Supplementing information to**

**Comammox *Nitrospira* act as key bacteria in weakly acidic soil via potential cobalamin sharing**

**Running Title:** The dominance of comammox *Nitrospira* in weakly acidic soil

Yuxiang Zhao^1,2^, Jiajie Hu^1^, Jiaqi Wang^1^, Xiangwu Yao^1^, Tong Zhang^3,4,5^, Baolan Hu^1,2,6#^

^1^Key Laboratory of Environment Remediation and Ecological Health, Ministry of Education, College of Environmental Resource Sciences, Zhejiang University, Hangzhou 310058, China

^2^College of Environmental and Resource Sciences, Zhejiang University, Hangzhou 310058, China

^3^Environmental Microbiome Engineering and Biotechnology Laboratory, Department of Civil Engineering, The University of Hong Kong, Hong Kong SAR 999077, China

^4^School of Public Health, The University of Hong Kong, Hong Kong SAR 999077, China

^5^Center for Environmental Engineering Research, The University of Hong Kong, Hong Kong SAR 999077, China

^6^Zhejiang Province Key Laboratory for Water Pollution Control and Environmental Safety, Hangzhou 310058, China

*Correspondence: blhu@zju.edu.cn (Baolan Hu)

## Supplementary results

### DNA stable-isotope probing (DNA-SIP) result

Results of AOA and AOB in DNA-SIP

After cultivation under low pH conditions, the peak positions of ammonia-oxidizing bacteria (AOB) in both the ^12^C group and the ^13^C group soil overlapped, indicating that AOB had little or no assimilation of ^13^CO_2_ (Figure S24, 25). In the ^13^C group, ammonia-oxidizing archaea (AOA) and comammox *Nitrospira* exhibited distinct single peaks at higher buoyant density fractions, suggesting that active AOA and comammox *Nitrospira* assimilated ^13^CO_2_ through autotrophic ammonia oxidation (Figure 5B, S26). The relative abundance of AOA and comammox *Nitrospira* in the light fractions of the ^13^C group was extremely low, indicating that AOA and comammox *Nitrospira* had replicated at least once and obtained "heavy" DNA, such that fragments not replicated were no longer detectable in the "light" DNA fractions. After cultivation under neutral pH conditions, compared to the ^12^C group, AOB, AOA, and comammox *Nitrospira* in the ^13^C group all exhibited distinct single peaks at higher buoyant density fractions, indicating that active AOB, AOA, and comammox *Nitrospira* assimilated ^13^CO_2_ through autotrophic ammonia oxidation (Figure S27). After cultivation under high pH conditions, compared to the ^12^C group, AOB in the ^13^C group exhibited a distinct single peak at higher buoyant density fractions, indicating that active AOB assimilated ^13^CO_2_ through autotrophic ammonia oxidation. In contrast, the peak positions of AOA and comammox *Nitrospira* in both the ^12^C control group and the ^13^C group overlapped, indicating that AOA and comammox *Nitrospira* had little or no assimilation of ^13^CO_2_. These results suggest that under low pH conditions, active ammonia-oxidizing bacteria are mainly AOA and comammox *Nitrospira*. AOB are mainly ammonia-oxidizing bacteria under high pH conditions. Under neutral pH conditions, the active ammonia-oxidizing bacteria are AOB, AOA, and comammox *Nitrospira*.

Both comammox *Nitrospira* clade A and clade B could be observed at all pH values (Figure S28). A unique comammox *Nitrospira* clade A branch exists in soils with pH as low as 3.5 and is evolutionarily separated from other sequences. This phenomenon was not found in comammox *Nitrospira* clade B. These results also confirmed our finding that only comammox *Nitrospira* clade A metagenome assembled genomes (MAGs) were obtained under low pH. Overall, these results showed that comammox *Nitrospira* clade A rather than clade B is the potential acidophilic cluster in soil.

Calculation for free ammonia

Free ammonia can be calculated using equation (8-1). C(NH_3_) represents the concentration of free ammonia (M). C(NH_4_^+^-N) represents the total ammonia/ammonium concentration (M). K_b_ is the dissociation constant of ammonia. Kw is the dissociation constant of water. *K_b_*/*K_w_* = *e^6.344/(273-T)^* (Table S11).

$C({NH}_{3})=\frac{\text{1}\text{0}^{\text{pH}}}{(K_{b}\text{/}\text{K}_{w}+\text{1}\text{0}^{\text{pH}})}\times C({NH}_{4}^{+}-N)$ （8-1）

Construction for the phylogenetic trees of active AOB, AOA and comammox *Nitrospira*

After annotated by Encyclopedia of Genes and Genomes (KEGG) Orthology (KO), we extracted sequences from metagenomic data annotated as "EC.1.14.99.39". These sequences represent potential *pmoA*, AOA, AOB, or comammox *Nitrospira amoA* genes. Further, we align these sequences with known ammonia-oxidizing microorganisms (AOMs) *amoA* gene sequences in MEGA7, followed by tree construction using the maximum likelihood method.

Phylogeny of terrestrial comammox *Nitrospira amoA* genes and its corresponding pH

We downloaded comammox *Nitrospira amoA* sequences from the NCBI database and observed their correlation with pH in the context of phylogenetic analysis. In detail, we employed the Biopython package to crawl 176 *amoA* sequences with the search query "*amoA* AND soil AND *Nitrospira* NOT genome". These sequences were then subjected to phylogenetic analysis alongside reference sequences of comammox *Nitrospira amoA* genes, with non-comammox *Nitrospira amoA* sequences excluded. Subsequently, the remaining sequences were compared again on NCBI to retrieve similar sequences and their associated metadata. We filtered for sequences sourced solely from soil and containing pH information, resulting in a final set of 272 comammox *Nitrospira amoA* sequences. Further, we align these sequences in MEGA7, followed by tree construction using the maximum likelihood method. Finally, pH information was appended to each sequence using iTOL v3 (Figure S29).

## Supplementary methods

### Sampling strategy

We selected forest, grassland, and cropland as three typical terrestrial ecosystems as these landuse type represent 78% of the global terrestrial ecosystems and 82% of the vegetated soil area in China [1,2]. These habitats are also the three most prominent soil types of interest [3]. In detail, forest is one of the largest terrestrial ecosystems on Earth, covering approximately 20% of China [1]. Grassland is another of the largest terrestrial ecosystems [4], covering approximately 30% of China's total land area [1]. Cropland is the most heavily human-impacted terrestrial ecosystem, covering approximately 18% of China's total land area [1] (Figure S1-S3 and Table S1). Soil samples with acidic pH (Group A) were primarily associated with forest and grassland land-use types, whereas neutral (Group B) and alkaline soils (Group C) were more frequently found in cropland and grassland, respectively (Table S2).

**The Relationship between various environmental factors and the abundance of comammox *Nitrospira***

Although it showed a significant correlation between pH and the abundance of comammox *Nitrospira*, we used various method to confirm their relationships, including random forest analysis (Figure S5), mantel test (Figure S6), hierarchical partitioning analysis (Figure S7), linear regression analysis (Table S4), variance partitioning analysis (VPA) (Table S5) and multiple linear regression model (Figure S8). Overall, all these analysis showed a similar results that pH was the main factor driving the relative abundance of comammox *Nitrospira*.

Random forest analysis

The main environmental factors for regulating the abundance of comammox *Nitrospira* were identified by a classification random forest analysis (Figure S5). In this random forest model, environmental factors served as predictors for the abundance of comammox *Nitrospira amoA*. To estimate the importance of these environmental factors, we used percentage increases in the mean squared error (MSE) of variables, with higher MSE% values implying variables of greater importance [5]. The significance of each predictor on the response variables was assessed with the “*rfPermute*” R package. 500 decision trees were set, followed by 1000 random permutations and 5 repetitions of 10-fold cross-validation.

Mantel test

We used Euclidean distances for environmental data and Bray-Curtis for the abundance of comammox *Nitrospira* (Figure S6)*.* We further computed partial Mantel correlations between the abundance of comammox *Nitrospira* and environmental data for each sample with the R packages “*vegan*” and “*ecodist*” (999 permutations).

Hierarchical partitioning analysis

Hierarchical partitioning analysis was carried out with the R package “*hier.part*” to quantify the amount of variation in the abundance of comammox *Nitrospira* explained by individual environmental factors (Figure S7).

Linear regression analysis in Table S3

We used linear regression analysis (comammox *Nitrospira* abundance = *a* + *b* × pH) to further confirm the relationship between the abundance of comammox *Nitrospira* (dependent variable) and pH (independent variable) in Table S4. The detailed structure is shown in Table S3. The linear regression analysis was carried out with the R package “*stats*”. pH was always set as the independent variable. The abundance of comammox *Nitrospira* was set as the dependent variable, respectively. “*a*” in the linear regression analysis refers to intercept, and “*b*” refers to slope.

VPA analysis in Table S4

To provide a quantification of the relative contribution of different environmental factors to the abundance of comammox *Nitrospira*, we performed a VPA model via the R package “*vegan*”. We calculated all possible combinations and the combination of the list 4 factors was the optimal combination. Explanation rate of variance was calculated for univariate, bivariate, trivariate, and all four variables (Table S5).

Multiple linear regression model

A multiple linear regression (MLG) model was constructed to reveal how pH and other environmental factors affected the abundance of comammox *Nitrospira.* In the MLG models, all the environmental factors were independent variables, and the abundance of comammox *Nitrospira* was dependent variable. We used R packages “*tidyverse*” and “*leaps*” to construct the MLR and obtain R^2^ for each model, respectively (Figure S8). Given the equal importance of optimal model and minimal model in the construction of MLG models, we further calculated the bayesian information criterion (bic) for the different models with a lower BIC indicating a better R^2^. Results of MLG model showed that although the R^2^ for the combination of “pH” and “Nitrite” was not the largest, they had the smallest BIC and was the simplest model, therefore this MLR was the most appropriate model. Furthermore, we calculated the contribution of “pH” and “Nitrite” to this model. Results highlighted the importance of pH, as it contributed 61.2% for the total model. Thus, pH is the main driving force contributed to the ecological success of comammox *Nitrospira*.

### Rarefaction analysis

The rarefaction analysis was performed based on the smallest number of sequences measured per sample (i.e., 14,527 reads per sample) (Figure S9), using the R packages “*vegan*”, “*doBy*”, and “*ggalt*”. The purpose of this analysis was to evaluate whether the sequencing depth was sufficient to capture the diversity of the bacterial communities. Both the observed OTU and α-diversity (Shannon index) curves per sample showed signs of leveling off, indicating that the sequencing depth of 14,527 reads per sample was adequate for the amplicon sequencing. Each sample was the mean of three technical replicates. The profiles of terrestrial bacterial communities at the genus level are shown in Figure S10.

### How to determined potential comammox *Nitrospira* ASVs

Even with the use of 16S rRNA gene sequencing it was difficult to distinguish comammox *Nitrospira* from traditional *Nitrospira*. Thus, we combined various methods to confirm the potential comammox *Nitrospira* amplicon sequence variants (PC ASVs), including phylogenetic analysis and random forest analysis.

Phylogenetic analysis

Since all comammox *Nitrospira* affiliated to *Nitrospira* Lineage II, phylogenetic analysis was used to differentiate *Nitrospira* ASVs that are affiliated with Lineage II. It was necessary to demonstrate whether the 16S rDNA V4 region (nearly 250 bp) was effective in recognizing ASVs affiliated to *Nitrospira* Lineage II. The sequences of the 16S rDNA V4 region from SILVA 138.1 were extracted using primersearch with the 515F’ (GTGCCAGCMGCCGCGGTAA) and 806R’ (GGACTACHVGGGTWTCTAAT) from SILVA 138.1. Only sequences from the known *Nitrospira* lineage were included in the phylogenetic analysis as root. MEGA7 was used to construct the phylogenetic tree and it was visualised in iTOL v3 (Figure S11). The result showed that 16S rDNA V4 region (~250 bp) could identify ASVs affiliated with Lineage II *Nitrospira* (Figure S11).

Random forest analysis

Since not all *Nitrospira* Lineage II ASVs are Comammox *Nitrospira*, we constructed a random forest model to identify the *Nitrospira* Lineage II ASVs most associated with changes in comammox *Nitrospira amoA* abundance to indentify the potential comammox (PC) ASVs. In this random forest model, *Nitrospira* Lineage II ASVs served as predictors for the abundance of comammox *Nitrospira amoA*. To estimate the importance of these environmental factors, we used percentage increases in the MSE of variables: higher MSE% values implied more important variables [5]. The significance of each predictor on the response variables was assessed with the “*rfPermute*” R package. 500 decision trees were set, followed by 1000 random permutations and 5 repetitions of 10-fold cross-validation. In the end, only 4 Lineage II *Nitrospira* ASVs were highly associated with the comammox *Nitrospira* *amoA* abundance (i.e., ASV 174, ASV 5493, ASV 6298, and ASV 2996) (Figure S12). These ASVs were the PC ASVs.

### rrn copy number for comammox *Nitrospira*, AOB and AOA.

To differ the rrn copy number for comammox Nitrospira, AOB and AOA, we extracted the rrn copy numbers of all AOB genera and *Nitrospira* detected in our samples from the Ribosomal RNA Operon Copy Number Database (rrndb). Due to the lack of high-throughput sequencing for archaea, we listed the rrn copy numbers of all AOA species included in rrndb. Results showed that the rrn copy number for all *Nitrospira* species (including comammox *Nitrospira*) was 1 (Table S8). The average rrn copy number of the detected AOB genera and all AOA species was 1.4 (Table S9–S10). Thus, the results of the rrn copy number suggest that the growth rate of comammox *Nitrospira* is slower, which is consistent with Costa predicted [6] and the affinity demonstrated by comammox *Nitrospira* [7]*.*

### Taxon/column shuffling null model

We employed the "taxon/column shuffling" null model, as recommended, to compute positive and negative cohesion to reflecting the potential microbial cooperation and competition [8]. In each iteration, we designated one taxon as the "focal taxon." For each taxon besides the focal taxon, abundances in the null matrix were permuted from their abundance distribution across all the samples. Then, we calculated Pearson correlations between the focal taxon and the randomized other taxa. This process of computing pairwise correlations between the focal taxon and all other taxa was repeated 999 times. The median correlations obtained from these 999 randomizations were considered the "expected" correlations for the focal taxon. We recorded the median value as the "expected" correlation, rather than the mean value, because the distributions were skewed toward larger values. Thus, a greater proportion of the distribution fell within one standard deviation of the median compared to within one standard deviation of the mean. We repeated this process for each taxon as the focal taxon, resulting in a matrix of expected taxon correlations. Finally, we subtracted the expected taxon correlations from their corresponding observed taxon correlations, thereby producing a matrix where each value represented the observed minus expected correlation for the given pair of taxa.

### The relationship between PC ASVs and potential bacterial interactions

We found that a decrease in pH could promote the bacterial cooperative potential (expressed as positive cohesion) (Figure S13, 14). Considering the dominance of comammox *Nitrospira* under low pH conditions, we further analyzed the association between cohesion cohesion and the relative abundance of PC ASVs. Significant correlations were observed between PC ASVs and positive cohesion (Figure 2C), rather than negative cohesion or sum of cohesion (Figure S15). Moreover, to determine whether this association was unique to PC ASVs or common to all *Nitrospira*, we analyzed the relationship between the relative abundance of total *Nitrospira* and total *Nitrospira* excluding PC ASVs in relation to pH, positive cohesion, and total cohesion (Figure S16). The results demonstrated that only PC ASVs exhibited significant correlations with positive cohesion. These findings suggest that PC ASVs uniquely contribute to promoting microbial interactions.

**Potential bacterial interactions**

Biotic interactions are an important component of deterministic community assembly [9,10]. Unraveling the significance of microbial interactions in contributing to ecological community assembly presents a significant challenge in ecology, especially in microbial ecology [11]. Despite the development of various theoretical approaches over the past century, there is currently no method available to precisely quantify the contributions of "microbial interaction" in complex or natural microbial communities. Statistical methods based on species co-occurrence and correlation are the most widely used approaches for discerning microbial interactions in community ecology. Despite the challenges associated with the use of co-occurrence analysis in microbial ecology studies, with careful application and thoughtful interpretation, it can still significantly contribute to ecological research. We employed various co-occurrence approaches (such as cohesion analysis and network analysis) to assess how comammox *Nitrospira* influence potential terrestrial bacterial interactions. To avoid over-interpretation and confusion, we cautiously describe the calculated correlations as “potential bacterial interactions”.

As both environment filtering and microbial interaction could cause the correlations between taxa, Goberna’s method was used to confirm the influence of various processes to the links between taxa (i.e. microbial interactions, environmental selecting, dispersal limitation and other processes) (Figure S17). Results showed that microbial interactions were the primary driver (94.4%), rather than environmental filtering (2.6%). Moreover, the link test for environmental filtering (LTEF) was further used to confirm the relationship between environmental filtering and the observed network links (Figure S18). We used |*r*| > 0.6 as cutoff and detected only < 3 % taxon-taxon-environment pairs among all the links. This showed that the links occurring in the network might be caused by real bacterial interactions. Overall, bacterial interactions were one of the main drivers to drive the bacterial community in our used network.

### How to determine comammox *Nitrospira* MAGs

After obtaining 11 *Nitrospira* MAGs, we used 2 processes to distinguish comammox *Nitrospira* MAGs from traditional *Nitrospira* MAGs.

The phylogenetic tree of all known *Nitrospira*

To reveal the phylogenetic placement of these MAGs within the Nitrospirae, 538 genomes from this phylum were downloaded from the NCBI-RefSeq database. 5 genomes from the phylum *Thermotogae* were treated as an outgroup. Thus, 554 genomes (543 download and 11 self made) were included in this analysis. We used Genome Taxonomy Database Toolkit (GTDB-Tk; v 2.3.0) to classify the taxonomic and phylogenetic analyses, and visualized in iTOL v3. We found that 3 MAGs could be attributed to comammox *Nitrospira* clade A (Figure S19).

The phylogenetic tree of *amoA* in the three comammox *Nitrospira* MAGs

*amoA*, *hao*, and *nxr* genes were observed in all three MAGs. We blasted the *amoA* genes of these MAGs against the NCBI database and found that they were similar to those of *Nitrospira sp. ENR4*, *Candidatus* Nitrospira kreftii and *Nitrospira sp. isolate bin 001*, respectively, all of which have been confirmed to be comammox *Nitrospira* MAGs (Figure S20). Overall, based on the phylogenetic tree of all known *Nitrospira* and *amoA*, we confirmed the 3 obtained MAGs belonged to comammox *Nitrospira*.

### Metabolic potential of terrestrial comammox *Nitrospira* MAGs

To further explore the presence of the proton motive force (PMF) efflux pump in these MAGs, we compared these MAGs to the structured antibiotic resistance genes database (SARG database) using diamond blastp (identity 80%, e-value 1e-7, cover 75%, aligned length 25). Remarkably, two set of efflux pump (MexEF-OprN, and MacAB-TolC) could be observed in these MAGs. Both MexEF-OprN and MacAB-TolC were PMF driven efflux pumps, whose running force is composed of the pH gradient and electrochemical potential inside and outside the cytoplasmic membrane [12]. Beyond the proton force, Bahram et al. highlighted the potential of fungal–bacterial antagonism to the bacterial community in soil, as fungi are well-known natural producers of many antibiotics [13]. In addition to antibiotics, MexEF-OprN could pump out aromatic hydrocarbons and quorum-sensing signaling molecules, and MacAB-TolC can extrude lipopolysaccharides and peptides as nonantibiotic resistance functions [14]. Therefore, in low pH soils where fungal antibiotics are present in large amounts, MexEF-OprN and MacAB-TolC could be encoded by comammox *Nitrospira* to counteract the stress of multiple antibiotics [15].

### Effect of outliers on the correlation between the relative abundance of comammox *Nitrospira* MAGs and pH

Considering the existence of outliers, we reconstructed a correlation analysis to reduce its impact on the statistical results. A clear correlation was observed, confirming that the relative abundance of comammox *Nitrospira* MAGs is indeed positively associated with pH (Figure S21).

### The effect of cobalamin

Many microorganisms require cobalamin to maintain various metabolic functions [16]. As a public good, cobalamin is notable because it could substantially affect microbial growth at low, even picomolar, external concentrations [17]. Cobalamin has also been shown to govern various of microbial processes, including the regulation of gene expression [18], replication and repair of DNA [19], tricarboxylic acid (TCA) cycle [20], biosynthesis of amino acid synthesis [21], and CO_2_ fixation [22].

### Three main cobalamin-dependent enzymes

Cobalamin auxotrophy is the most common form of vitamin auxotrophy in marine plankton [23]. Six enzymes require cobalamin as a cofactor, and these central enzymes include methionine synthase (*metH*), methylmalonyl-CoA mutase (*mutA*), and ribosomal small subunit methyltransferase (*rsmB*), as methyl transfer and rearrangement reactions are the main processes driven by cobalamin.

*metH*

Cobalamin-dependent methionine synthase catalyzes the transfer of a methyl group from N5-methyltetrahydrofolate to homocysteine, producing tetrahydrofolate and methionine [21]. Cobalamin-dependent methionine synthesis (*metH*) was present in at least 80% of the microbes, while Cobalamin-independent methionine synthesis (*metE*) was only present in 30%. Moreover, most microbes can utilize the more efficient Cobalamin-dependent methionine synthesis pathway (*metH*) when Cobalamin is available, as opposed to the less efficient Cobalamin-independent pathway [21].

*mutA* and *rsmB*

This enzyme catalyzes the reversible isomerization of L-methylmalonyl-CoA to succinyl-CoA using 5’-deoxyadenosyl radical, produced by cobalamin, as a cofactor participating in the generation of radicals that allow isomerization of the substrate [21].The metabolic pathways of the methyl donor required for rsmB's function in RNA methylation are cobalamin-dependent.

### Abundance of cobalamin synthesis genes in different pH groups

To confirm the results obtained by MAGs (Figure 4, S22), contigs data (after de-duplication and length filtering, see Section Method 3.5) was used to analyze cobalamin synthesis genes. Contigs longer than 2000 bp were retained because most cobalamin synthesis genes are in the range of 1000-1500 bp. Contigs longer than 2000 bp help us better identify its taxonomy. These data were also used as the basis for obtaining the MAGs, allowing for the distinction of which contigs belong to the comammox MAGs. After obtaining the Open Reading Frames (ORFs) using Prodigal, KofamScan was used to annotate the functional roles of each ORF. The abundance of each contigs (TPM, Transcripts Per Million) were obtained via coverm.

Results showed that the pathway of Step A (Corring ring aerobic biosynthesis), Step B (Final synthesis and repair) and Step C (Dimethylbenzimidazole synthesis) were complete. Although more phylum were involved in anaerobic biosynthetic at the contig level, it remained incomplete, which was consistent with the results from MAGs (Figure S23A). Both *Proteobacteria* phylum and comammox *Nitrospira* possessed the complete aerobic cobalamin biosynthetic pathway. In weakly acidic soil (Group A, pH < 6.5), comammox *Nitrospira* contributed 48.5-99.1% (mean 76.0%) to the remaining steps of Step A (Corrin ring aerobic biosynthesis), except for the processes Uroporphyrinogen III → Precorrin-2 and HBA → HBA a,c-diamide (Figure S23B). This is because the genes involved in these two steps were present in both anaerobic and aerobic biosynthetic pathways. In neutral and alkaline soils, *Proteobacteria* became the major potential supplier of cobalamin, contributing 68.4%, while comammox contributed 5.1% (Figure S23B). More than six phyla encoded complete metabolic pathways for Step B and Step C, including *Actinobacteriota,* *Nitrospirota* (including comammox *Nitrospira*), *Halobacteriota*, *Proteobacteria*, *Methylomirabilota* and *Thermoproteota.* These results were consistent with the results obtained by MAGs (Figure 4) and previous study in soil [24]. Overall, the results obtained from the analysis based on contigs were consistent with those of MAGs, which showed that comammox *Nitrospira* was one of the dominant providers of cobalamin in weakly acidic soil (Figure S23).

**REFERENCES**

1. Tang, Xuli, Xia Zhao, Yongfei Bai, Zhiyao Tang, Wantong Wang, Yongcun Zhao, Hongwei Wan et al. 2018. “Carbon pools in china's terrestrial ecosystems: new estimates based on an intensive field survey.” *Proceedings of the National Academy of Sciences of the United States of America* 115: 4021–4026. http://doi.org/10.1073/pnas.1700291115

2. Smith, Pete, Helmut Haberl, Alexander Popp, Karl-Heinz Erb, Christian Lauk, Richard Harper, Francesco N. Tubiello et al. 2013. “How much land-based greenhouse gas mitigation can be achieved without compromising food security and environmental goals?” *Global Change Biology* 19: 2285–2302. http://doi.org/10.1111/gcb.12160

3. Chu, Haiyan, Gui-Feng Gao, Yuying Ma, Kunkun Fan, Manuel Delgado-Baquerizo. 2020. “Soil microbial biogeography in a changing world: recent advances and future perspectives.” *Msystems* 5:00803–19. http://doi.org/10.1128/mSystems.00803-19

4. Nan, Zhibiao. 2005. “The grassland farming system and sustainable agricultural development in china.” *Grassland Science* 51: 15–19. http://doi.org/10.1111/j.1744-697X.2005.00003.x

5. Trivedi, Pankaj, Manuel Delgado-Baquerizo, Chanda Trivedi, Hangwei Hu, Ian C. Anderson, Thomas C. Jeffries, Jizhong Zhou, Brajesh K. Singh. 2016. “Microbial regulation of the soil carbon cycle: evidence from gene-enzyme relationships.” *ISME Journal* 10: 2593–2604. http://doi.org/10.1038/ismej.2016.65

6. Costa, Engracia, Julio Perez, Jan-Ulrich Kreft. 2006. “Why is metabolic labour divided in nitrification?” *Trends in Microbiology* 14: 213–219. http://doi.org/10.1016/j.tim.2006.03.006

7. Kits, K. Dimitri, Christopher J. Sedlacek, Elena V. Lebedeva, Ping Han, Alexandr Bulaev, Petra Pjevac, Anne Daebeler et al. 2017. “Kinetic analysis of a complete nitrifier reveals an oligotrophic lifestyle.” *Nature* 549: 269–272. http://doi.org/10.1038/nature23679

8. Herren, Cristina M., Katherine D. Mcmahon. 2017. “Cohesion: a method for quantifying the connectivity of microbial communities.” *ISME Journal* 11: 2426–2438. http://doi.org/10.1038/ismej.2017.91

9. D'Amen, Manuela, Heidi K. Mod, Nicholas J. Gotelli, Antoine Guisan. 2018. “Disentangling biotic interactions, environmental filters, and dispersal limitation as drivers of species co-occurrence.” *Ecography* 41: 1233–1244. http://doi.org/10.1111/ecog.03148

10. Barner, Allison K., Kyle E. Coblentz, Sally D. Hacker, Bruce A. Menge. 2018. “Fundamental contradictions among observational and experimental estimates of non-trophic species interactions.” *Ecology*. 99: 557–566. http://doi.org/10.1002/ecy.2133

11. Zhou, Jizhong, Daliang Ning. 2017. “Stochastic community assembly: does it matter in microbial ecology?” *Microbiology and Molecular Biology Reviews* 81:00002–17. http://doi.org/10.1128/MMBR.00002-17

12. Klenotic, Philip A., Mitchell A. Moseng, Christopher E. Morgan, Edward W. Yu. 2021. “Structural and functional diversity of resistance-nodulation-cell division transporters.” *Chemical Reviews* 121: 5378–5416. http://doi.org/10.1021/acs.chemrev.0c00621

13. Bahram, Mohammad, Falk Hildebrand, Sofia K. Forslund, Jennifer L. Anderson, Nadejda A. Soudzilovskaia, Peter M. Bodegom, Johan Bengtsson-Palme et al. 2018. “Structure and function of the global topsoil microbiome.” *Nature* 560: 233–237. http://doi.org/10.1038/s41586-018-0386-6

14. Henderson, Peter J. F., Claire Maher, Liam D. H. Elbourne, Bart A. Eijkelkamp, Ian T. Paulsen, Karl A. Hassan. 2021. “Physiological functions of bacterial "multidrug" efflux pumps.” *Chemical Reviews* 121: 5417–5478. http://doi.org/10.1021/acs.chemrev.0c01226

15. Liu, Zishu, Yuxiang Zhao, Baofeng Zhang, Jiaqi Wang, Lizhong Zhu, Baolan Hu. 2023. “Deterministic effect of pH on shaping soil resistome revealed by metagenomic analysis.” *Environmental Science & Technology* 57: 985–996. http://doi.org/10.1021/acs.est.2c06684

16. Shelton, Amanda N., Erica C. Seth, Kenny C. Mok, Andrew W. Han, Samantha N. Jackson, David R. Haft, Michiko E. Taga. 2019. “Uneven distribution of cobamide biosynthesis and dependence in bacteria predicted by comparative genomics.” *ISME Journal* 13: 789–804. http://doi.org/10.1038/s41396-018-0304-9

17. Croft, Martin. T., Andrew D. Lawrence, Evelyne Raux-Deery, Martin J. Warren, Alison G. Smith. 2005. “Algae acquire vitamin B_12_ through a symbiotic relationship with bacteria.” Nature 438: 90–93. http://doi.org/10.1038/nature04056

18. Manuel Ortiz-Guerrero, Juan, Maria Carmen Polanco, Francisco J. Murillo, S. Padmanabhan, Montserrat Elias-Arnanz. 2011. “Light-dependent gene regulation by a coenzyme B_12_-based photoreceptor.” *Proceedings of the National Academy of Sciences of the United States of America* 108: 7565-7570. http://doi.org/10.1073/pnas.1018972108

19. Blakley, Raymond. L., Horace Albert Barker. 1964. “Cobamide stimulation of reduction of ribotides to deoxyribotides in *Lactobacillus leichmannii*.” *Biochemical and Biophysical Research Communications* 16: 391–397. http://doi.org/10.1016/0006-291X(64)90363-8

20. Bertrand, Erin M., Andrew E. Allen. 2012. “Influence of vitamin B auxotrophy on nitrogen metabolism in eukaryotic phytoplankton.” *Frontiers in Microbiology* 3: 375. http://doi.org/10.3389/fmicb.2012.00375

21. Banerjee, Ruma V., Rowena G. Matthews. 1990. “Cobalamin-dependent methionine synthase.” *Faseb Journal* 4: 1450-1459. http://doi.org/10.1096/fasebj.4.5.2407589

22. Berg, Ivan A., Daniel Kockelkorn, Wolfgang Buckel, Georg Fuchs. 2007. “A 3-hydroxypropionate/4-hydroxybutyrate autotrophic carbon dioxide assimilation pathway in archaea.” *Science* 318: 1782-1786. http://doi.org/10.1126/science.1149976

23. Croft, Martin T., Martin J. Warren, Alison G. Smith. 2006. “Algae need their vitamins.” *Eukaryotic Cell* 5: 1175-1183. http://doi.org/10.1128/EC.00097-06

24. Lu, Xinda, Katherine R. Heal, Anitra E. Ingalls, Andrew C. Doxey, Josh D. Neufeld. 2020. “Metagenomic and chemical characterization of soil cobalamin production.” *ISME Journal* 14: 53-66. http://doi.org/10.1038/s41396-019-0502-0

25. Goberna, Marta, Alicia Montesinos-Navarro, Alfonso Valiente-Banuet, Yannick Colin, Alicia Gomez-Fernandez, Santiago Donat, Jose A. Navarro-Cano, Miguel Verdu. 2019. “Incorporating phylogenetic metrics to microbial co-occurrence networks based on amplicon sequences to discern community assembly processes.” *Molecular Ecology Resources* 19: 1552-1564. http://doi.org/10.1111/1755-0998.13079


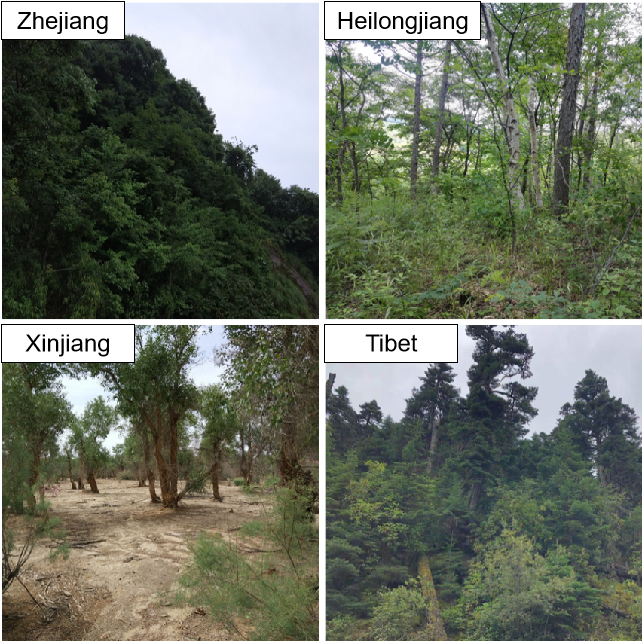


**Figure S1 Sampling sites of forest soils.**


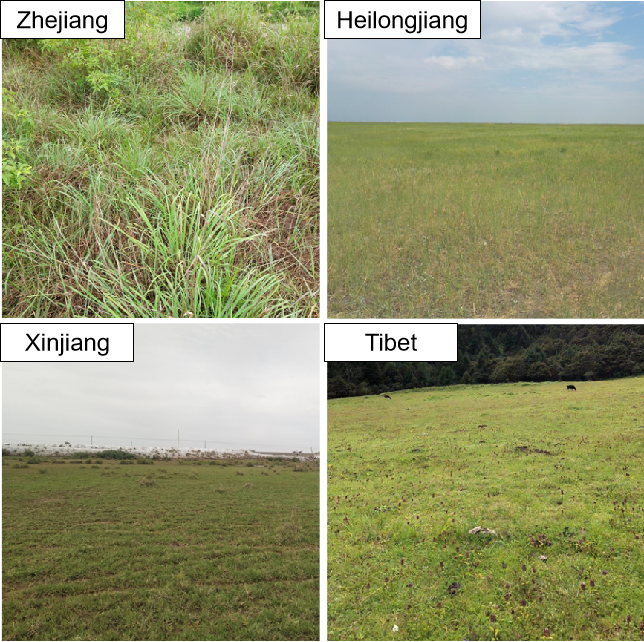


**Figure S2 Sampling sites of grassland soils.**


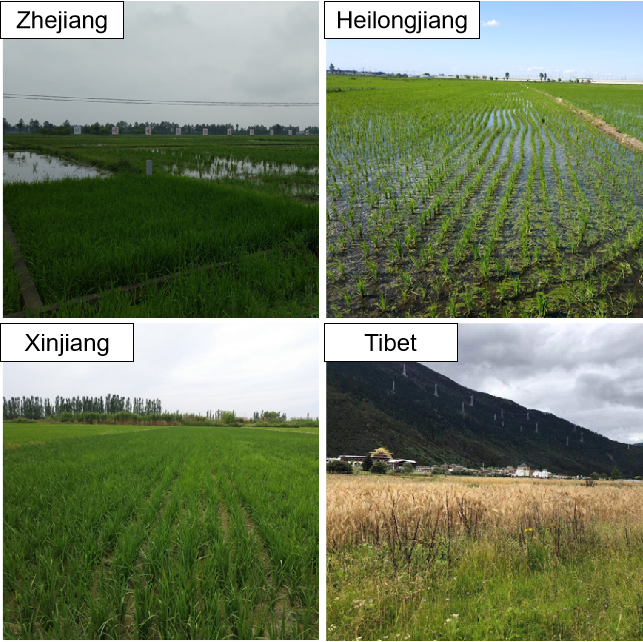


**Figure S3 Sampling sites of cropland soils**


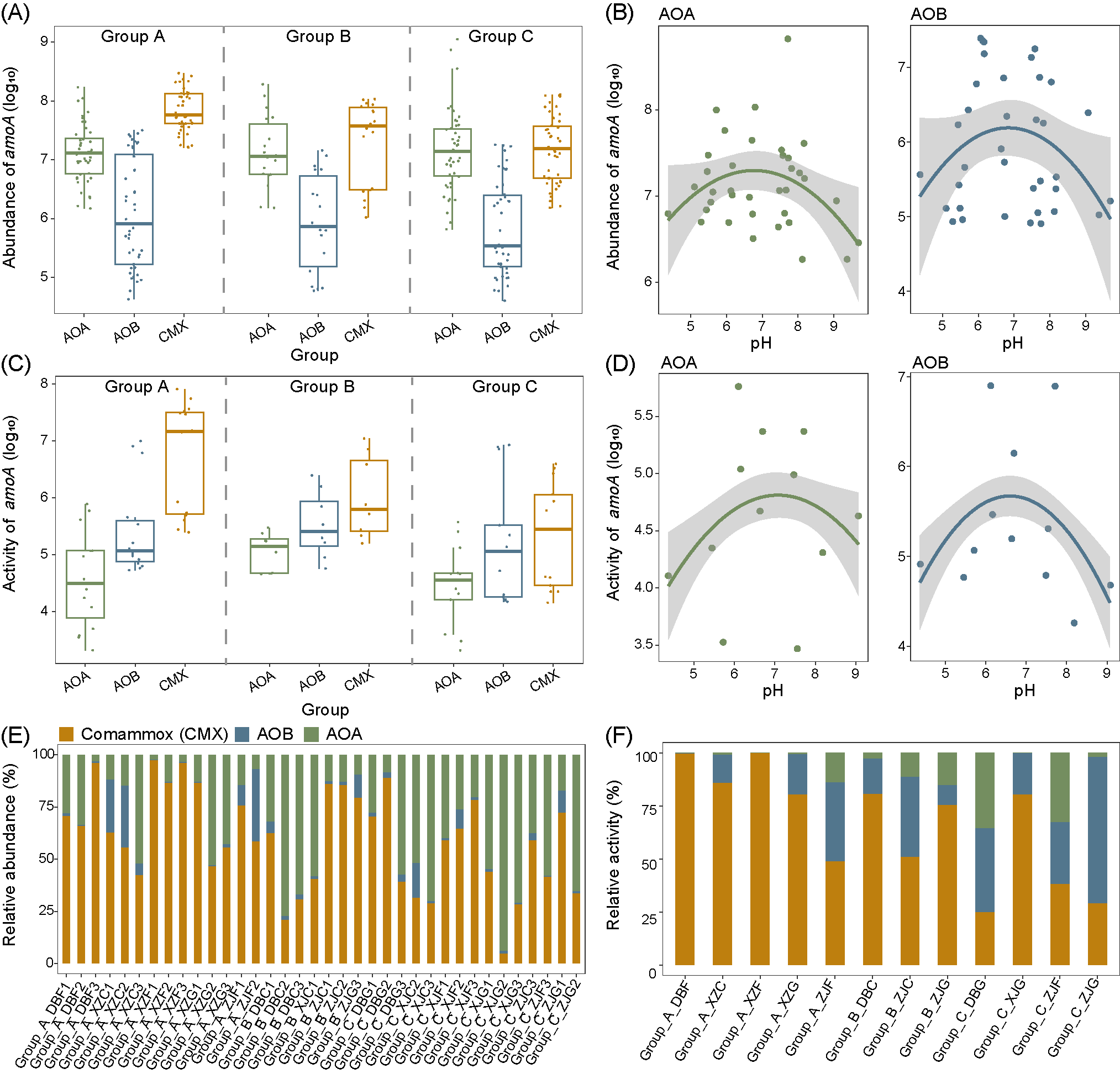


**Figure S4 The abundance and activity of ammonia oxidizers.** (A) The abundance of different ammonia oxidizers. (B) The relationship between the abundance of ammonia-oxidizing archaea (AOA) and ammonia-oxidizing bacteria (AOB) and pH. (C) The activity of different ammonia oxidizers. (D) The relationship between the activity of AOA and AOB and pH. (E) The relative abundance of different ammonia oxidizers. Relative abundance refers to the percentage of *amoA* abundance of each ammonia oxidizer in a sample, relative to the total *amoA* abundance of all ammonia oxidizers in that sample. (F) The relative activity of different ammonia oxidizers. Relative activity refers to the percentage of *amoA* activity of each ammonia oxidizer in a sample, relative to the total *amoA* activity of all ammonia oxidizers in that sample.
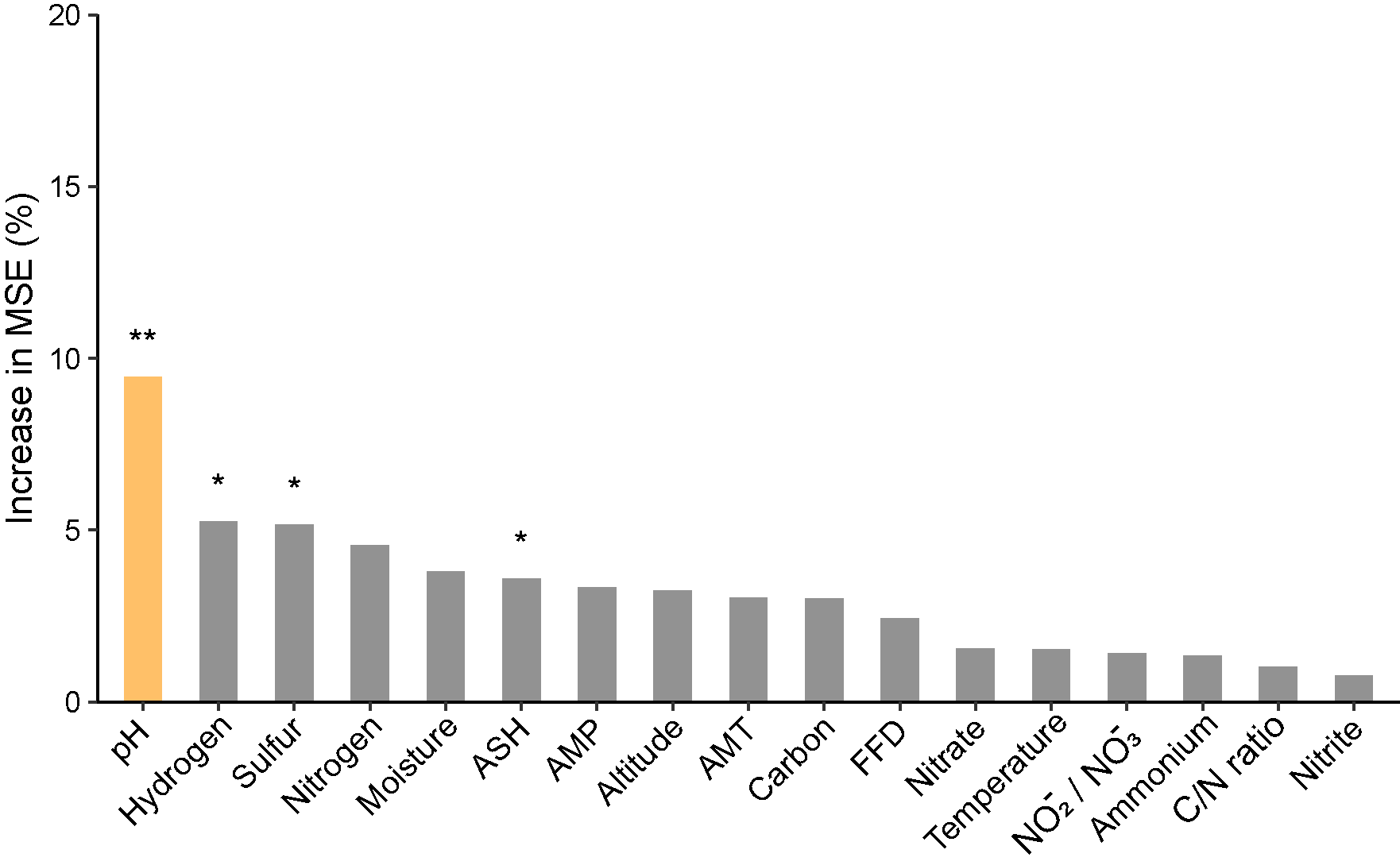


**Figure S5 Random forest analysis.** Unadjusted *p* values of the one-way ANOVA are labeled as** when *p* < 0.01, * when *p* < 0.05.


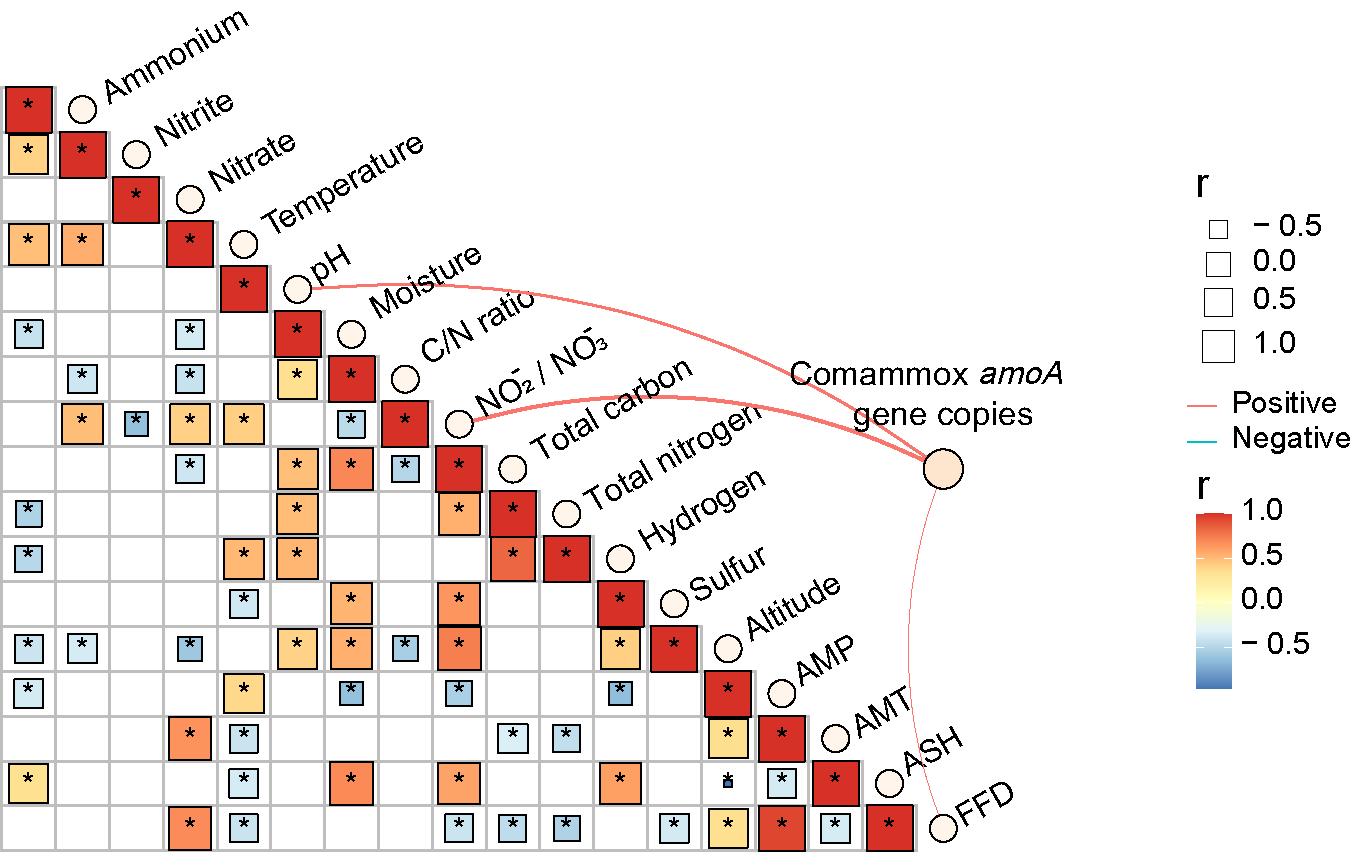


**Figure S6 Mantel test.** Pairwise comparisons of environmental factors are shown in the triangle, with the colour gradient and square size denoting Pearson’s correlation coefficient. The line size indicates the Mantel’s r statistic between each environmental factor and the abundance of comammox *Nitrospira amoA*. The color indicates positive (red) and negative (blue) correlation.


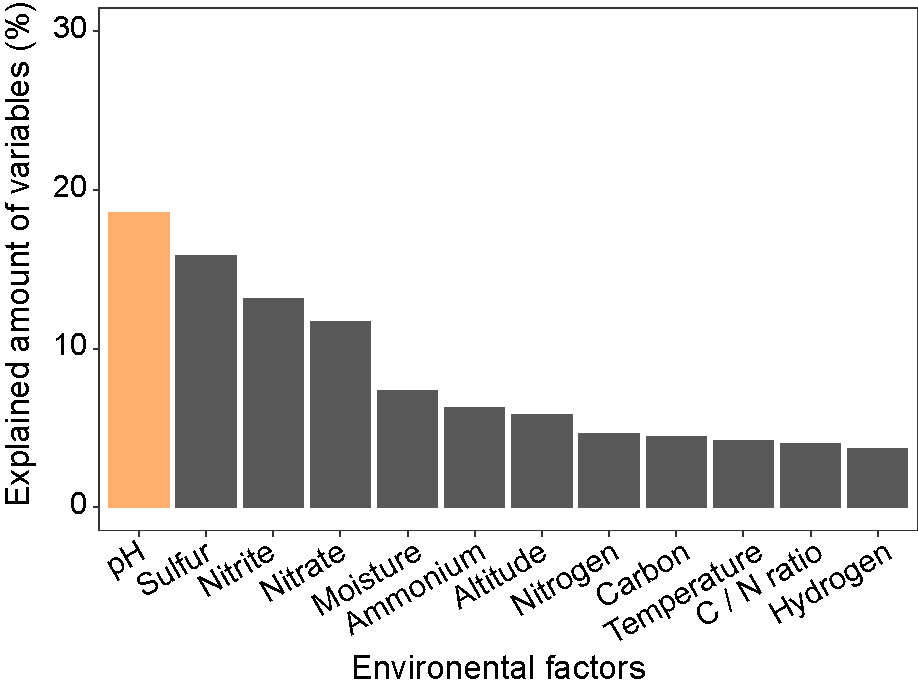


**Figure S7 Hierarchical partitioning analysis.** pH was highlighted in orange.


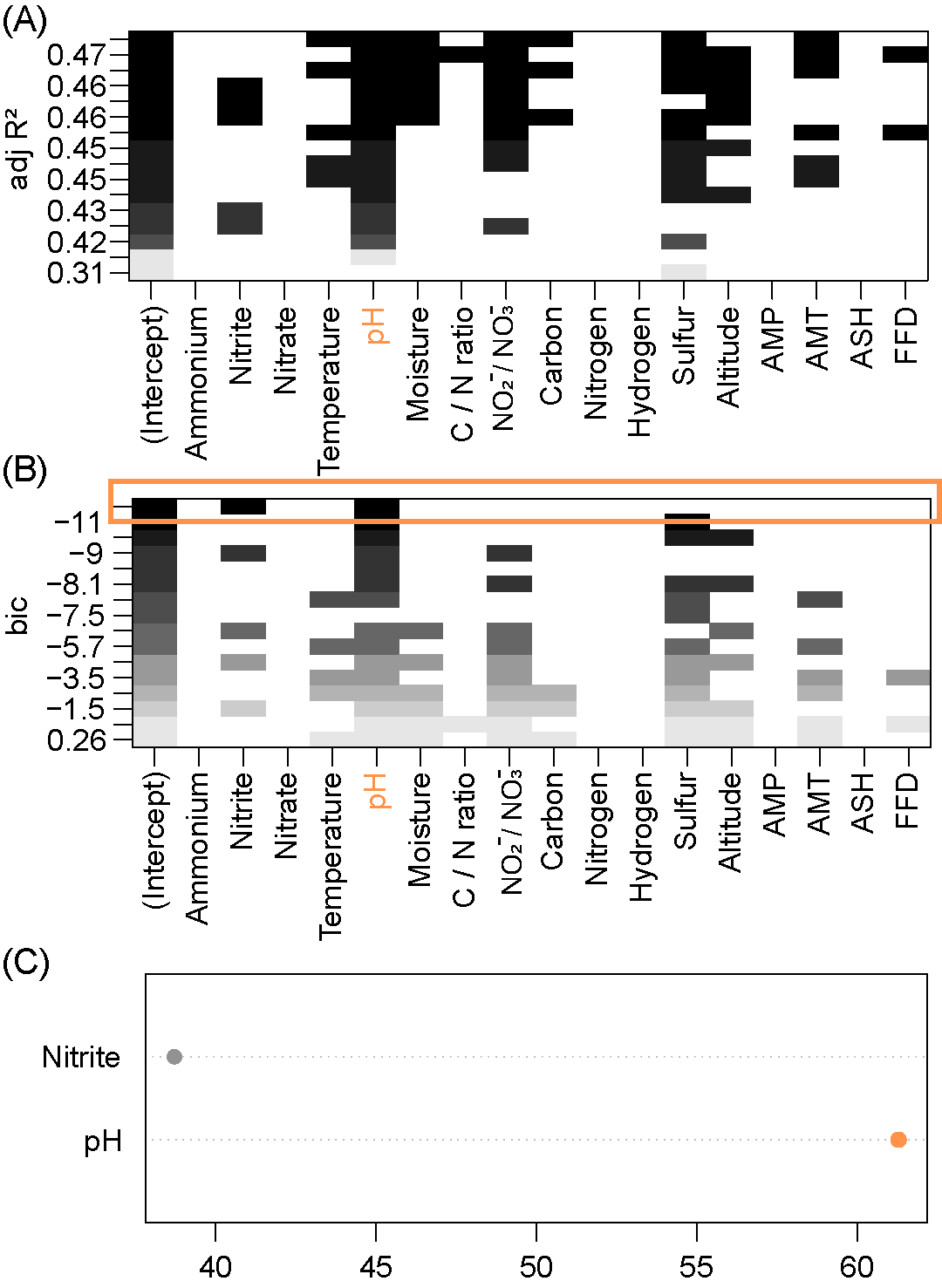


**Figure S8 Multiple linear regression between mean copy number (MCN) and environmental factors.** (A) R^2^ for different models. (B) Bayesian Information Criterion (bic) for different models. (C) Relative importance of predictor variables. The orange box indicates the selected model.


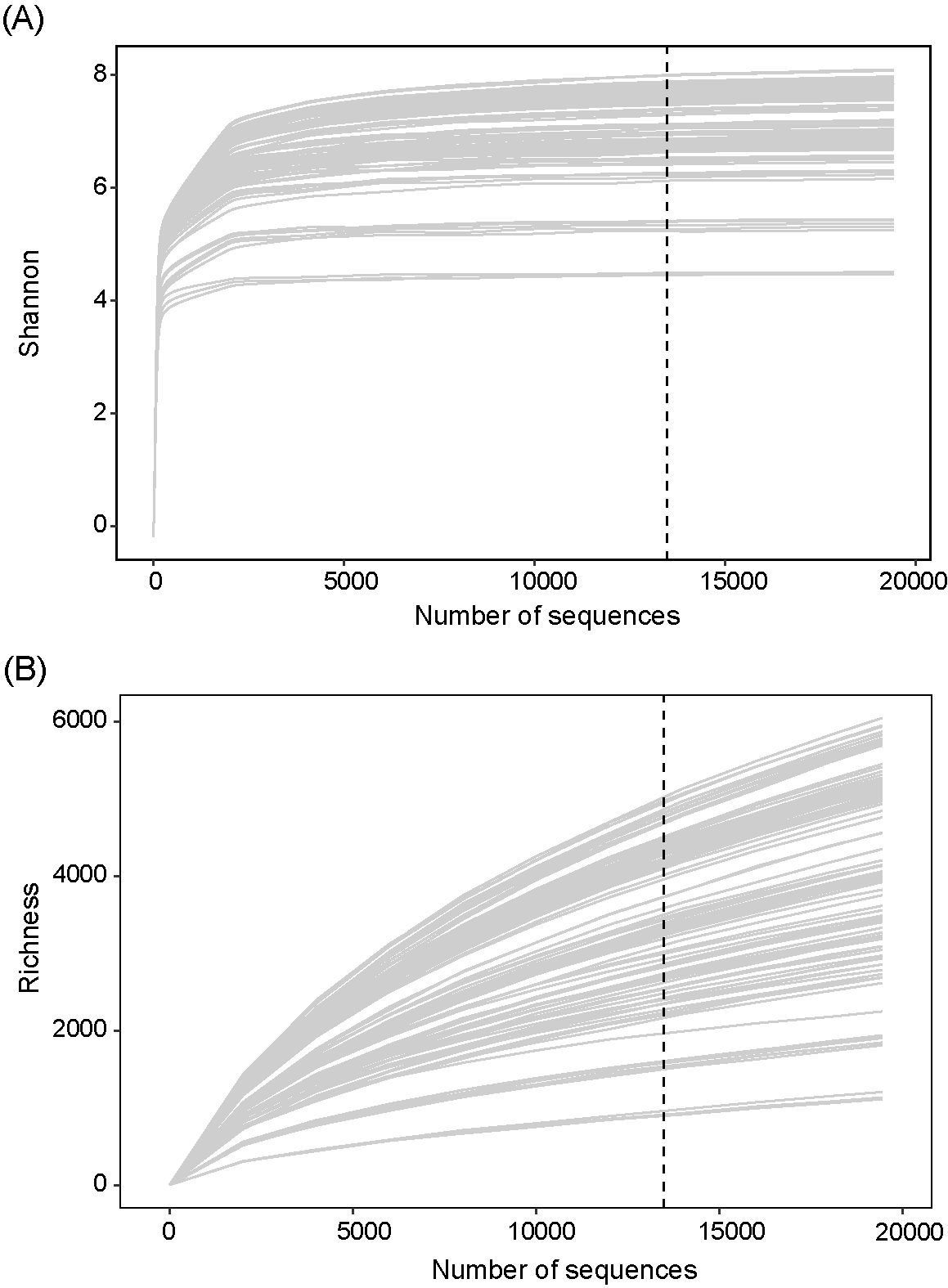


**Figure S9 Rarefaction curves.** (A) The α-diversities per sample (Shannon index). (B) The richness per sample (Shannon index). The α-diversities were calculated based on ASV data. Vertical dash line indicates 14,527 reads which was smallest number of sequences per sample.


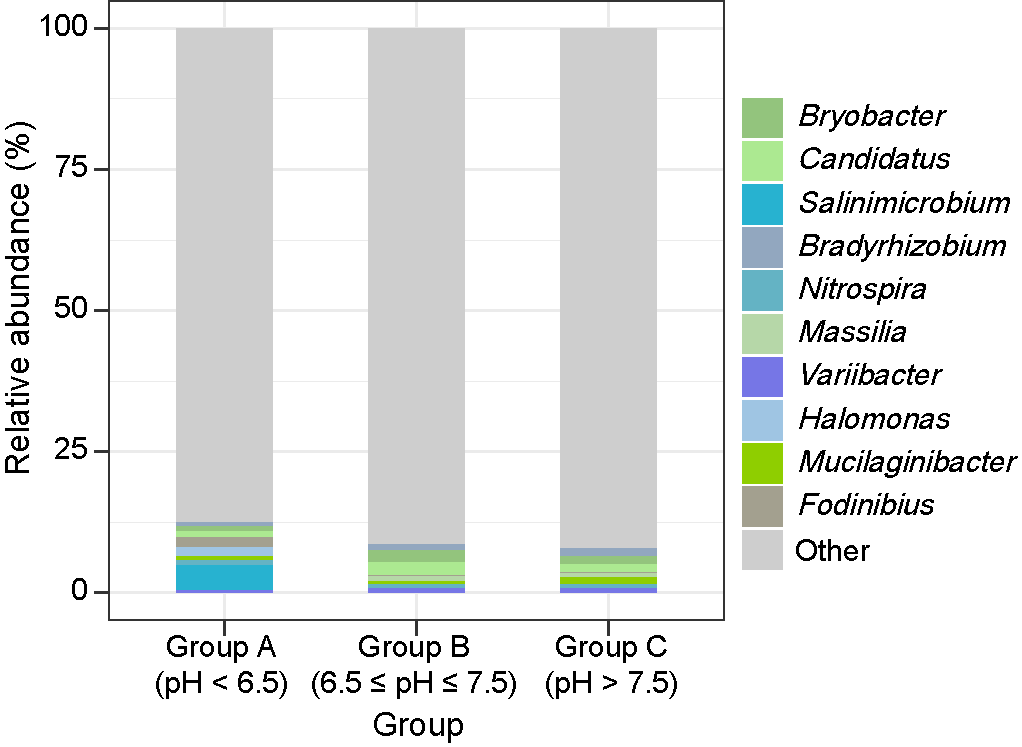


**Figure S10 The overall community structure of the soil bacteria with 10 most abundant taxa at the genus level.**

*
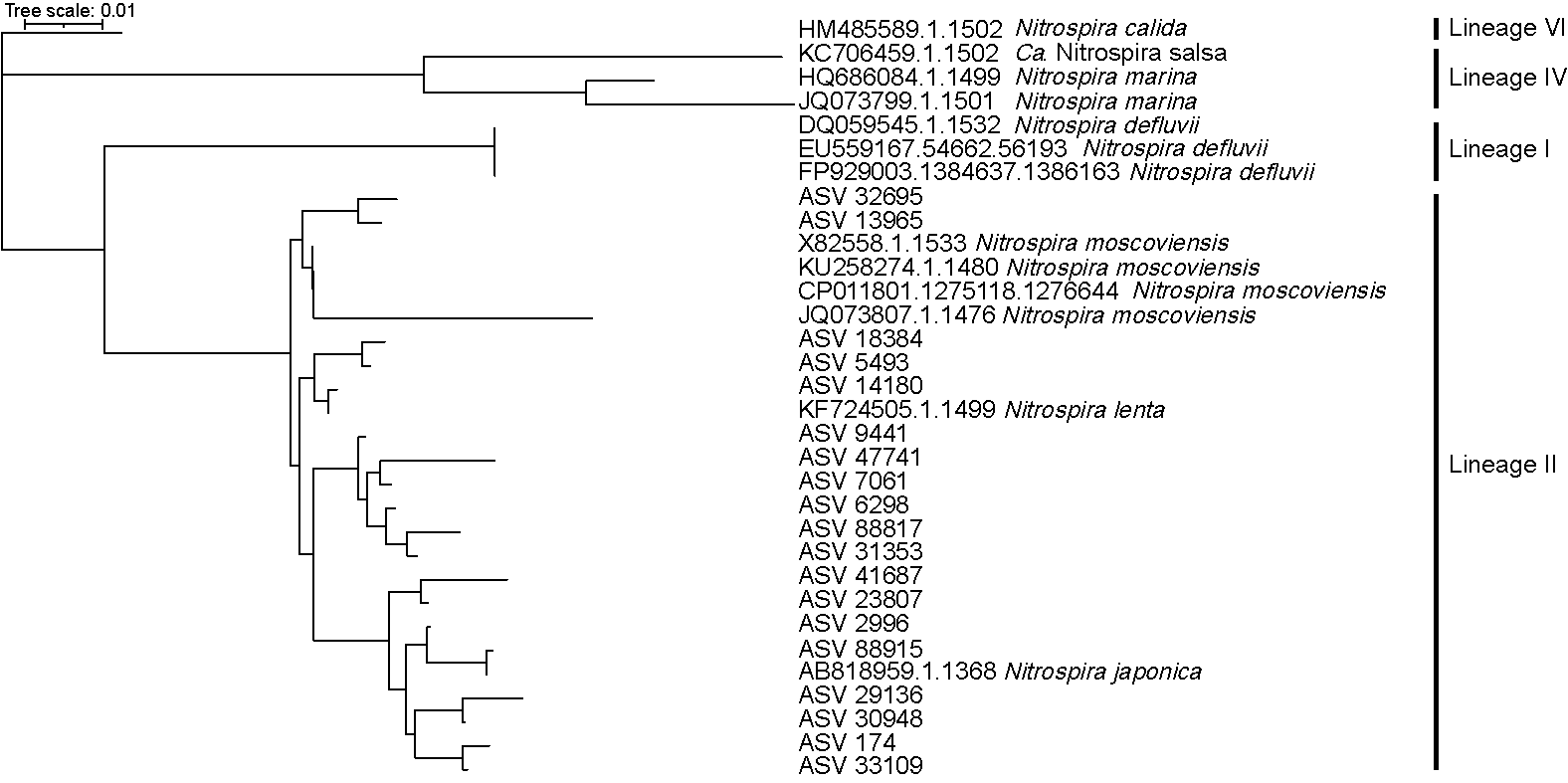
*

**Figure S11 Phylogenetic analysis of the *Nitrospira* Lineage II amplicon sequence variants (ASVs).** The ASV were obtained from the high-throughput sequencing. Species for each Lineage were obtained from SILVA 138.1.


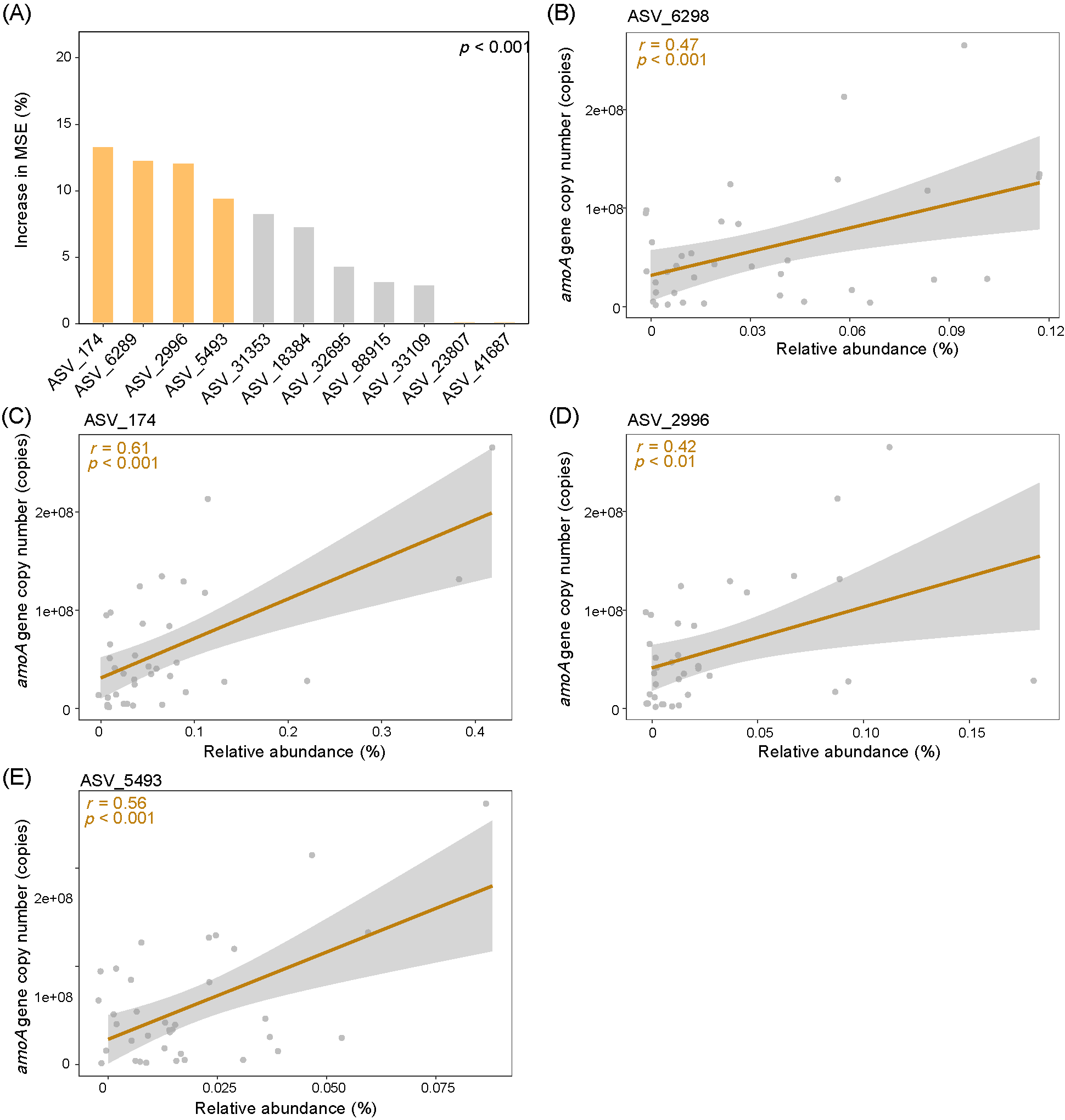


**Figure S12 The screening for potential comammox *Nitrospira* ASVs (PC ASVs).** (A) The random forest model analysis to predict the potential comammox *Nitrospira* ASVs. *Nitrospira* Lineage II ASVs served as predictors for the abundance of comammox *Nitrospira amoA*. (B) The relationship between ASV_6298 and *amoA* gene copy number. (C) The relationship between ASV_174 (PC ASVs) and *amoA* gene copy number. (D) The relationship between ASV_2996 and *amoA* gene copy number. (E) The relationship between ASV_5493 and *amoA* gene copy number. Gray shaded areas in B, C, and D indicate 95% confidence interval. MSE, mean squared error.**
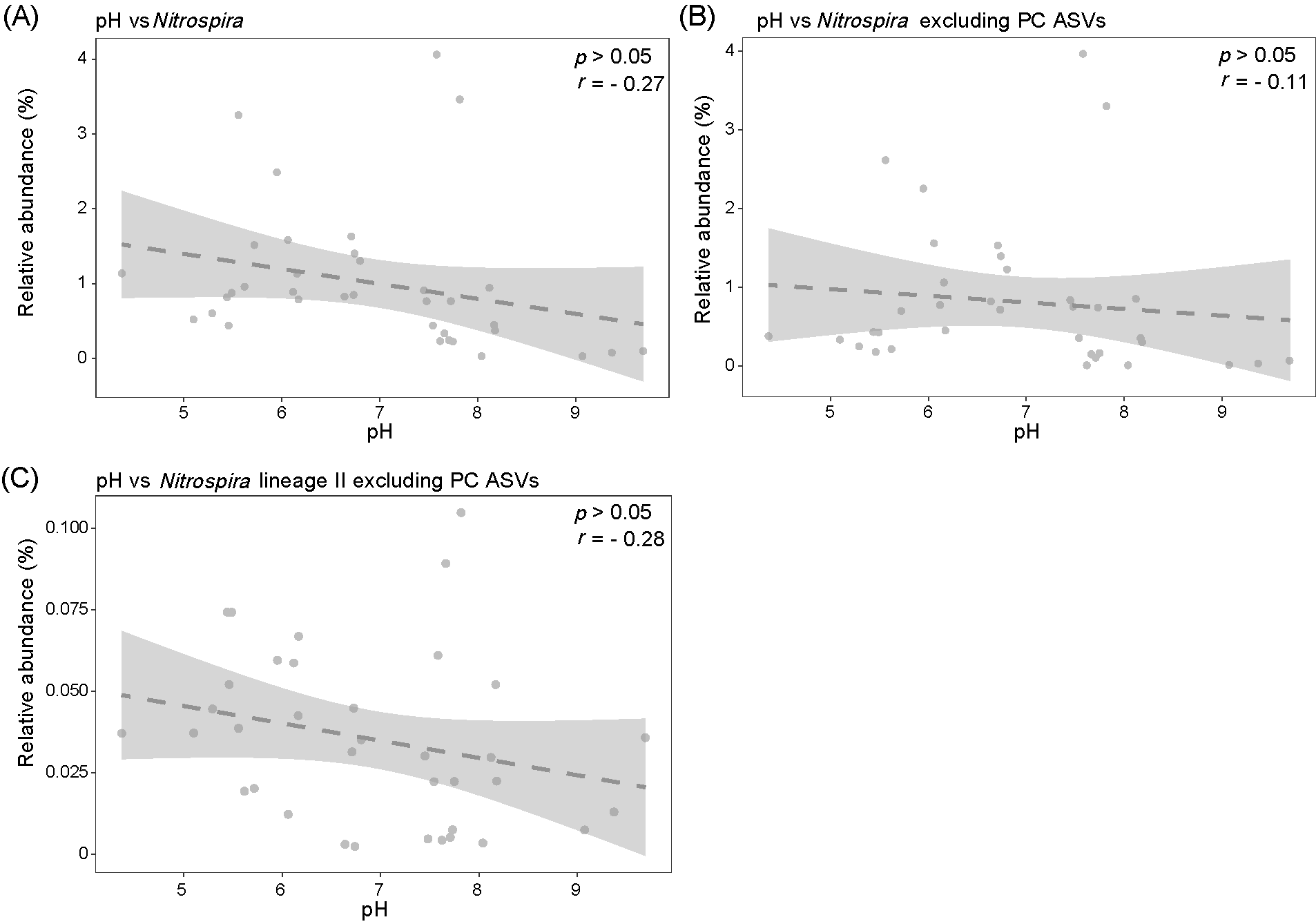
Figure S13 The relationship between total *Nitrospira* and *Nitrospira* excluding PC ASVs and pH.** (A) The relationship between the relative abundance of total *Nitrospira* and pH. Total *Nitrospira* indicated the relative abundance of *Nitrospira* genus. (B) The relationship between the relative abundance of *Nitrospira* excluding PC ASVs and pH. “*Nitrospira* excluding PC ASVs” refers to the total relative abundance of *Nitrospira* minus the relative abundance of PC ASVs. (C) The relationship between the relative abundance of *Nitrospira* Lineage II excluding PC ASVs and pH. “*Nitrospira* Lineage II excluding PC ASVs” refers to the relative abundance of *Nitrospira* Lineage II minus the relative abundance of PC ASVs.


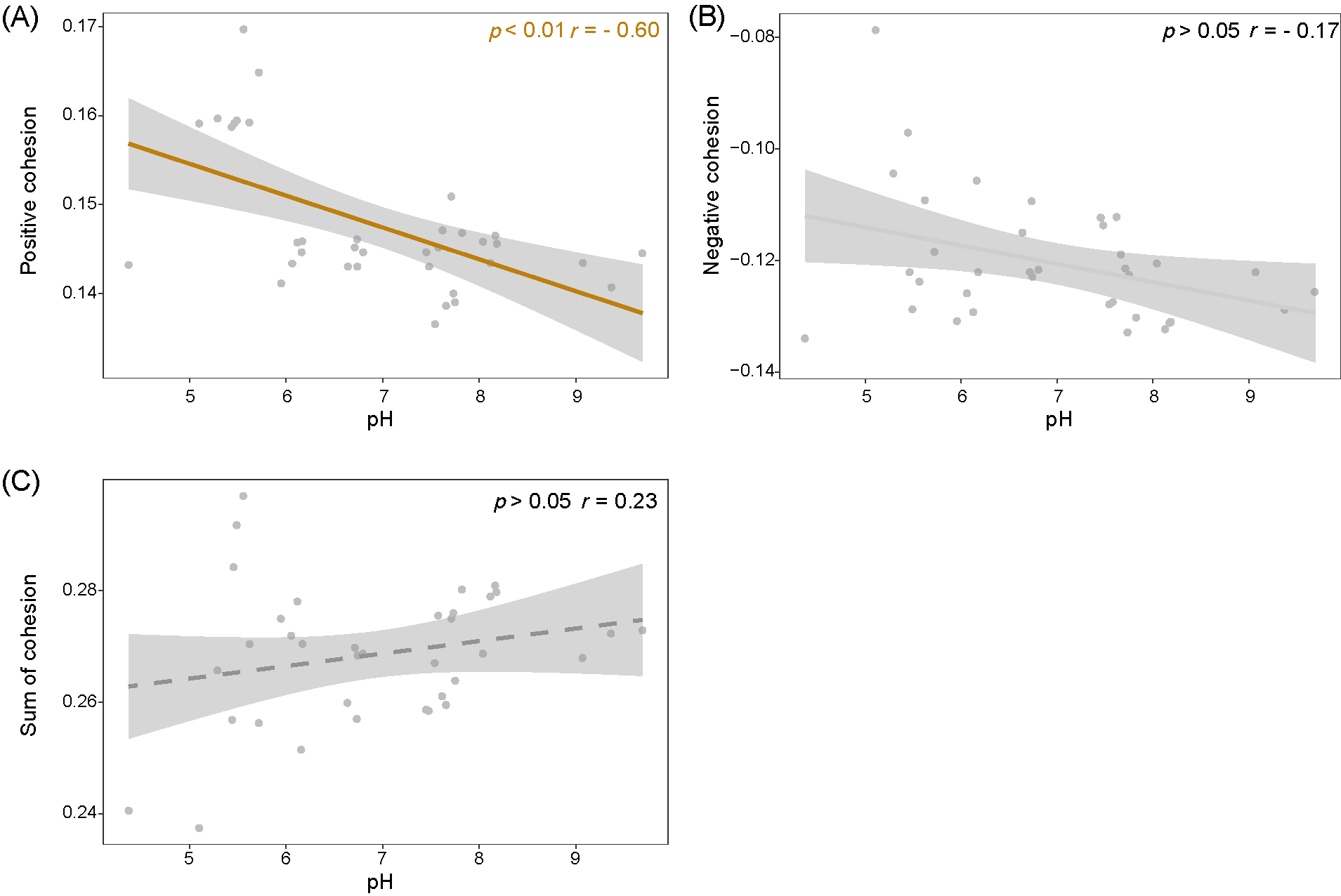


**Figure S14 The relationship between positive cohesion, negative cohesion and sum of cohesion and pH.** (A) The relationship between pH and positive cohesion. (B) The relationship between pH and negative cohesion. (C) The relationship between pH and sum of cohesion. The sum of cohesion is calculated as the absolute value of the negative cohesion, added to the sum of the positive cohesion.


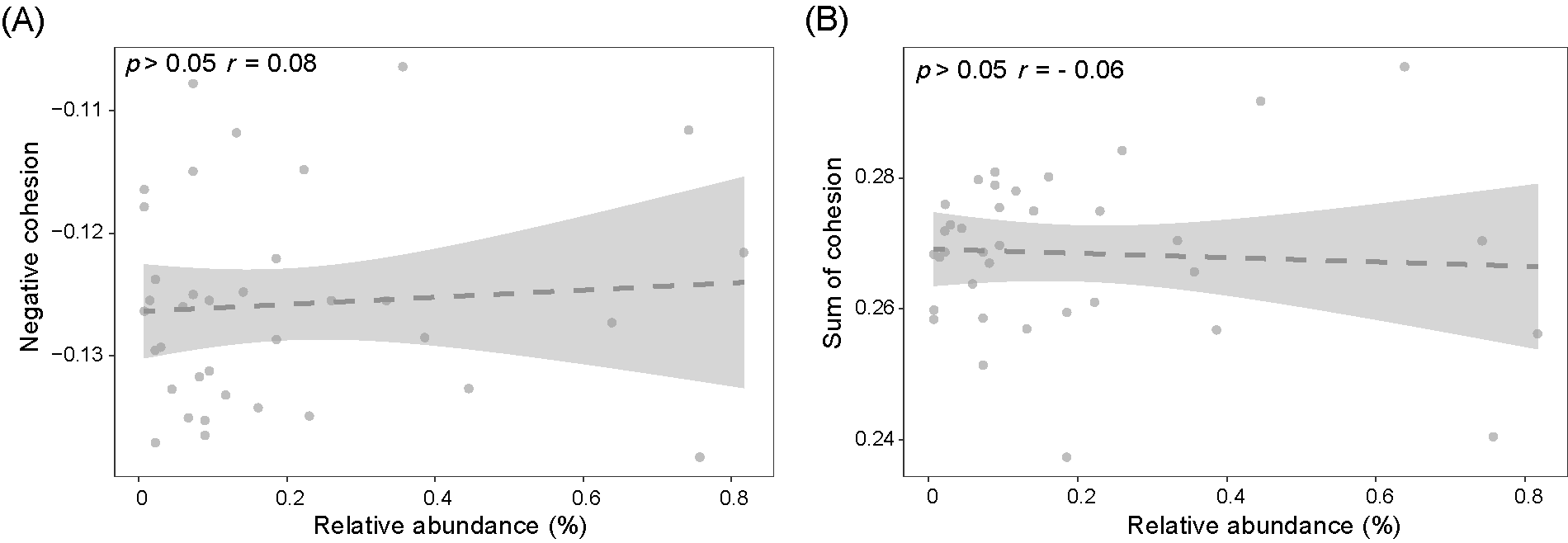


**Figure S15 The relationship between PC ASVs and negative cohesion and sum of cohesion.** (A) The relationship between the relative abundance of PC ASVs and negative cohesion. (B) The relationship between the relative abundance of PC ASVs and sum of cohesion. The sum of cohesion is calculated as the absolute value of the negative cohesion, added to the sum of the positive cohesion.


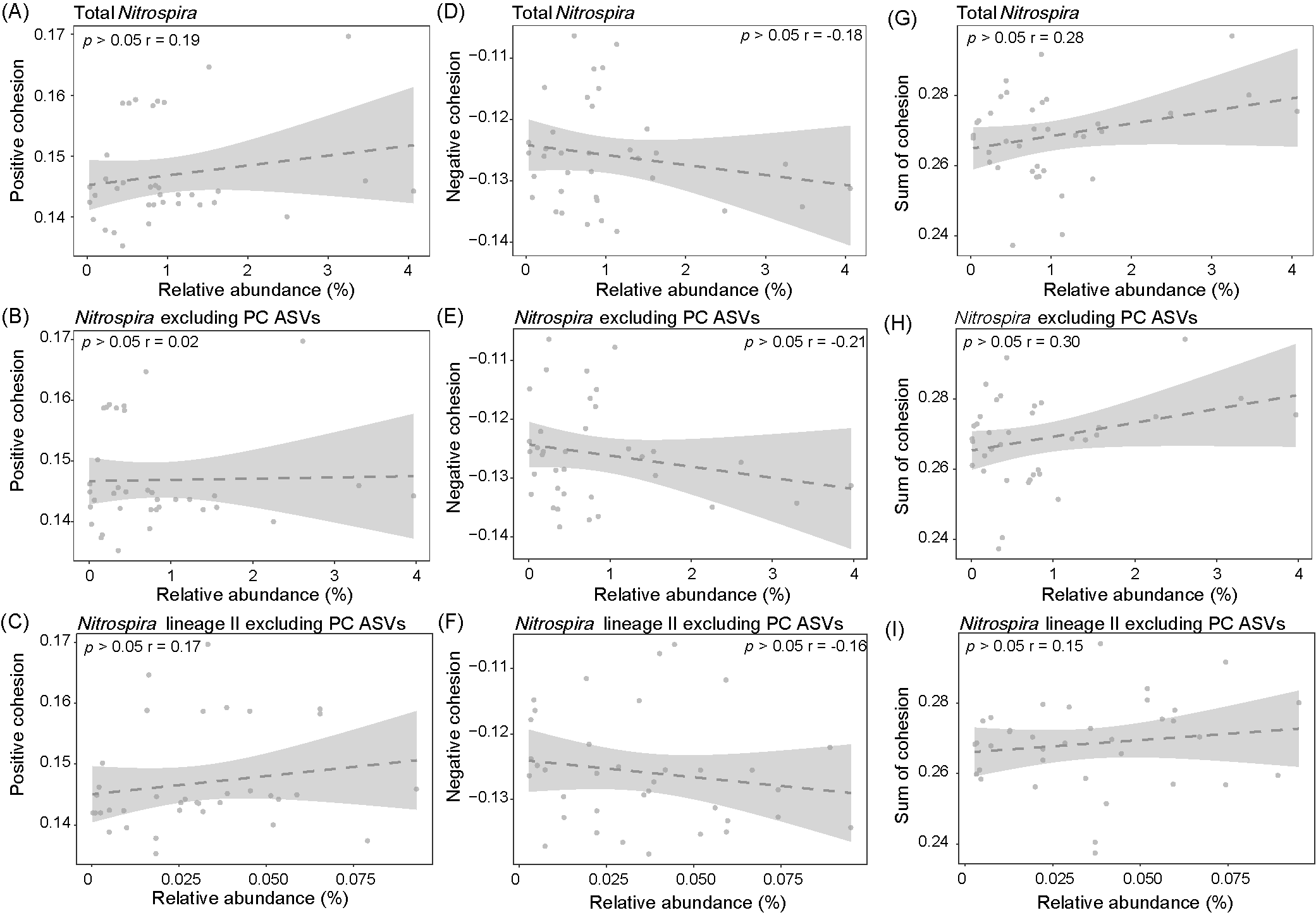


**Figure S16 The relationship between positive cohesion, negative cohesion and sum of cohesion and *Nitrospira*.** (A) The relationship between the relative abundance of total *Nitrospira* and positive cohesion. (B) The relationship between the relative abundance of *Nitrospira* excluding PC ASVs and positive cohesion. (C) The relationship between the relative abundance of *Nitrospira* lineage II excluding PC ASVs and positive cohesion. (D) The relationship between the relative abundance of total *Nitrospira* and negative cohesion. (E) The relationship between the relative abundance of *Nitrospira* excluding PC ASVs and negative cohesion. (F) The relationship between the relative abundance of *Nitrospira* lineage II excluding PC ASVs and negative cohesion. (G) The relationship between the relative abundance of total *Nitrospira* and sum of cohesion. (H) The relationship between the relative abundance of *Nitrospira* excluding PC ASVs and sum of cohesion. (I) The relationship between the relative abundance of *Nitrospira* lineage II excluding PC ASVs and sum of cohesion. The sum of cohesion is calculated as the absolute value of the negative cohesion, added to the sum of the positive cohesion. Total *Nitrospira* indicated the relative abundance of *Nitrospira* genus. “*Nitrospira* excluding PC ASVs” refers to the total relative abundance of *Nitrospira* minus PC ASVs. “*Nitrospira* Lineage II excluding PC ASVs” refers to the relative abundance of *Nitrospira* Lineage II minus PC ASVs.


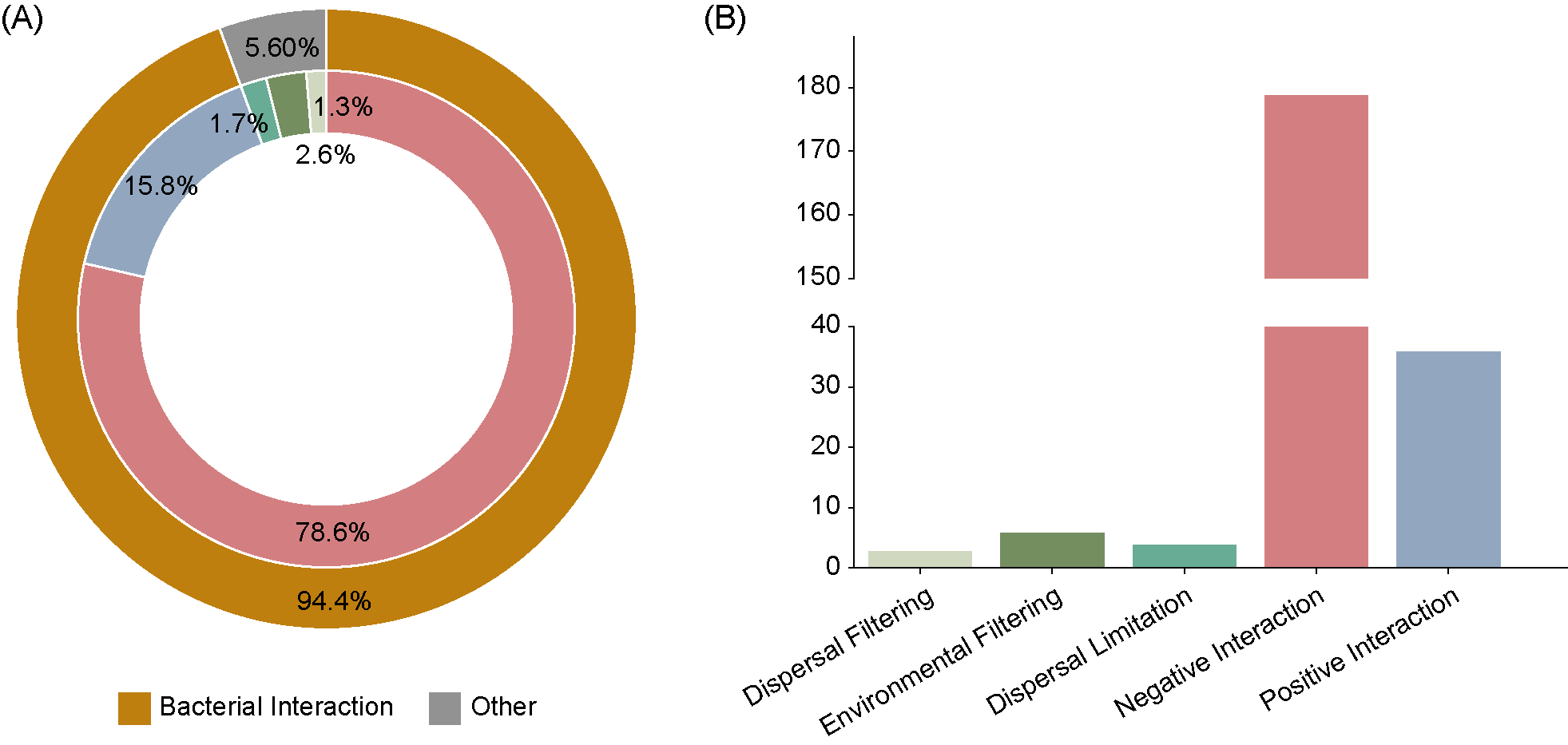


**Figure S17 Quantification of the relative contribution of community assembly processes to molecular ecological network analysis (MENA) links.** The tool was reported in Goberna et. al 2019 [25]. In detail, the process contained three processes: I) constructed the co‐occurrence networks with set group, II) attribution of co-occurring patterns to assembly processes using spatial and environmental data, and III) calculation and analyzation of the phylogenetic distances between aggregated (co-occurring) or segregated (co-excluding) species. (A) The relative contribution of each community assembly process to the observed links. (B) The number of links associated with each community assembly process.


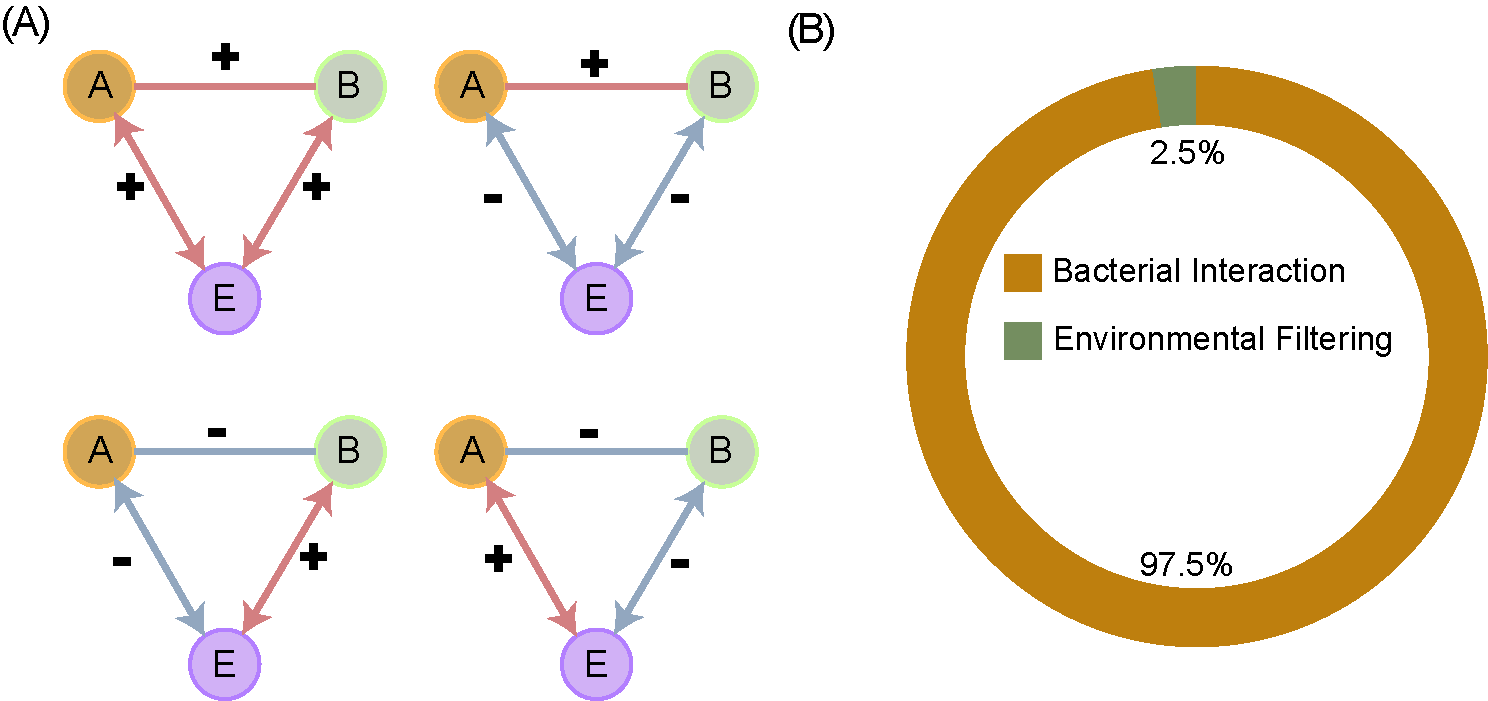


**Figure S18 Link Test for Environmental filtering (LTEF) for disentangling the contributions of environmental filtering to the observed network links.** (A) The framework to screen the potential taxon-taxon-environment links. A and B indicated the nodes (i.e., various taxa). E was the environmental factors with the same dimension as the taxa. Correlations between A-E as well as B-E were calculated based on the inter-domain network, which focused on the cross-domain associations between taxa and environment and eliminated links between taxa and taxa or environmental factor and environmental factor. Moreover, Both A-E and B-E should be significantly (*p* < 0.05) correlated with correlation strengths above a set threshold |*r*|. (B) The results of LTEF analysis.


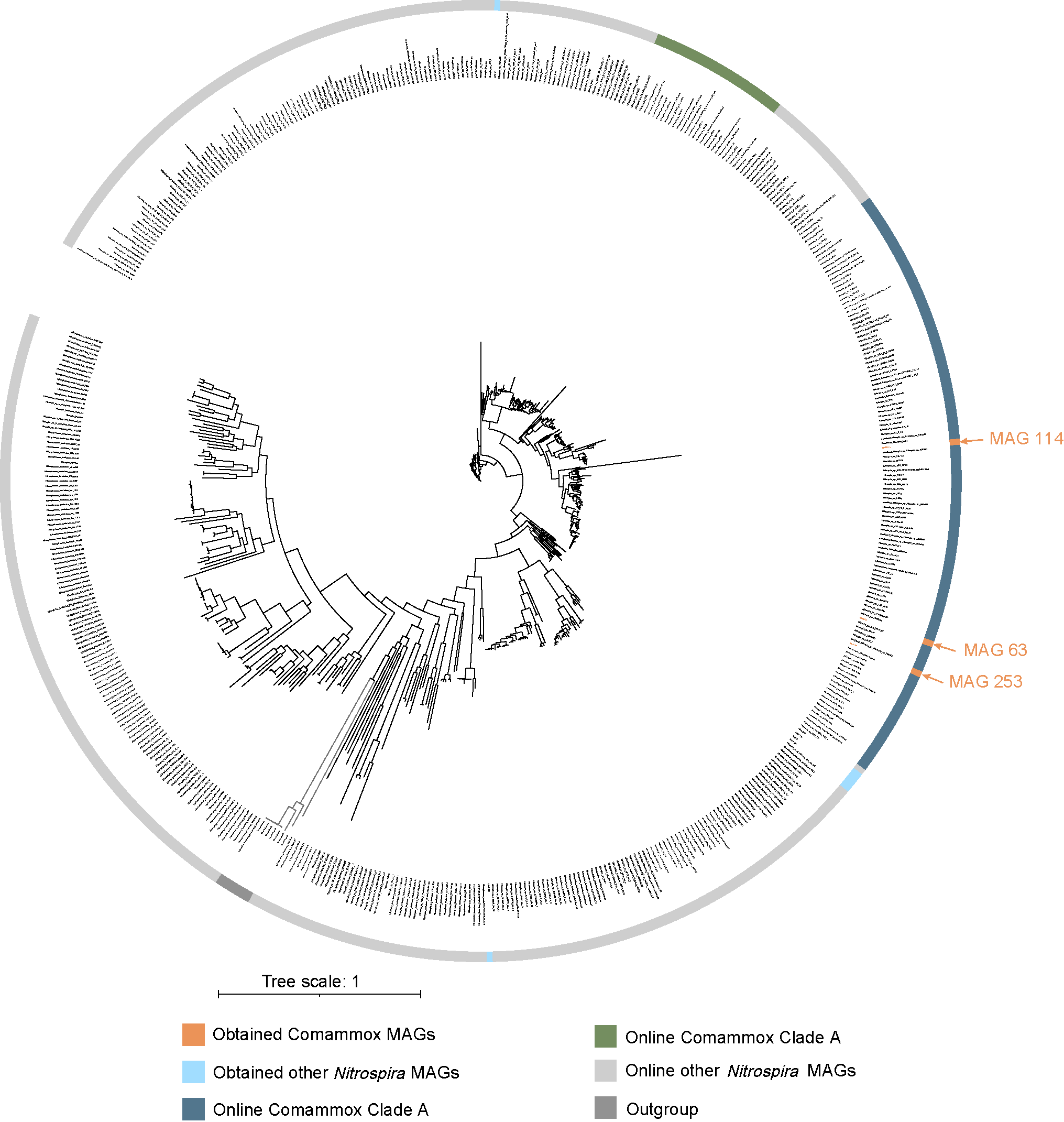


**Figure S19 The phylogenetic tree of all known *Nitrospira*.** The MAG labeled in orange are the potential comammox *Nitrospira* obtained in this study.


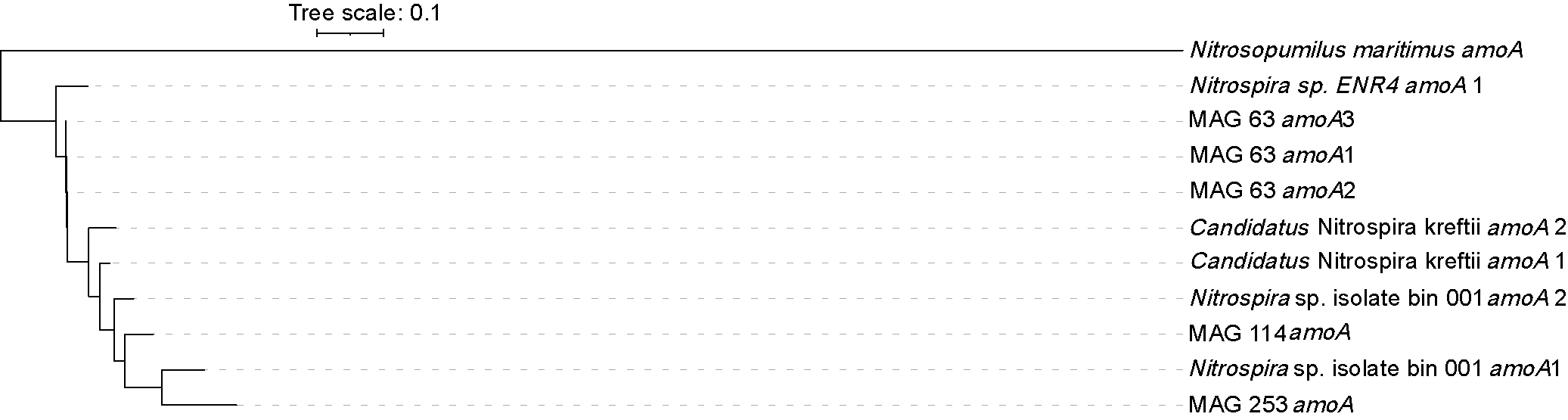


**Figure S20 The phylogenetic tree of *amoA* gene.**


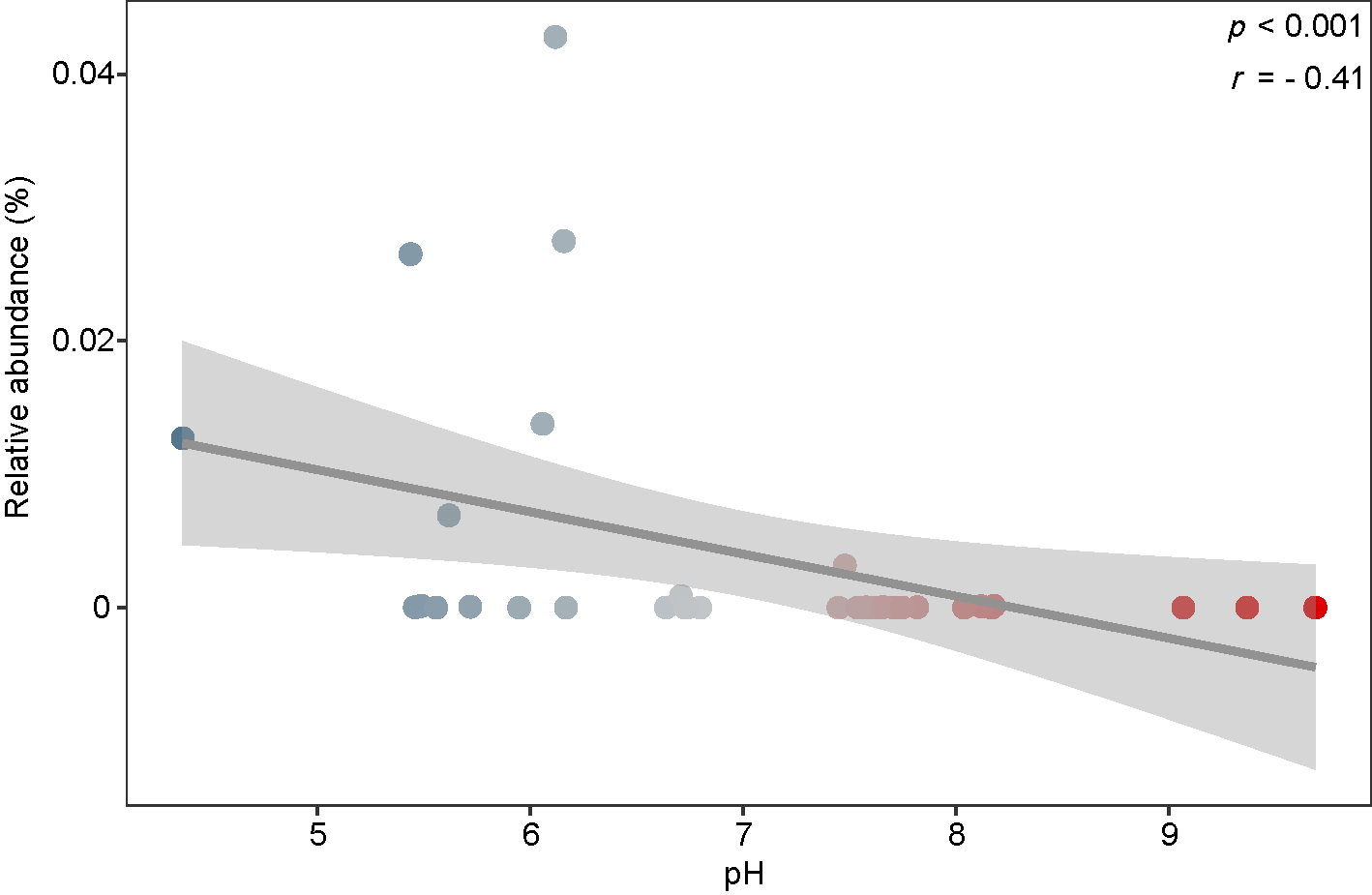


**Figure S21 Relationship between pH and the relative abundance of commamox *Nitrospira* MAGs excluding outliers.** Gray shaded area indicates 95% confidence intervals.


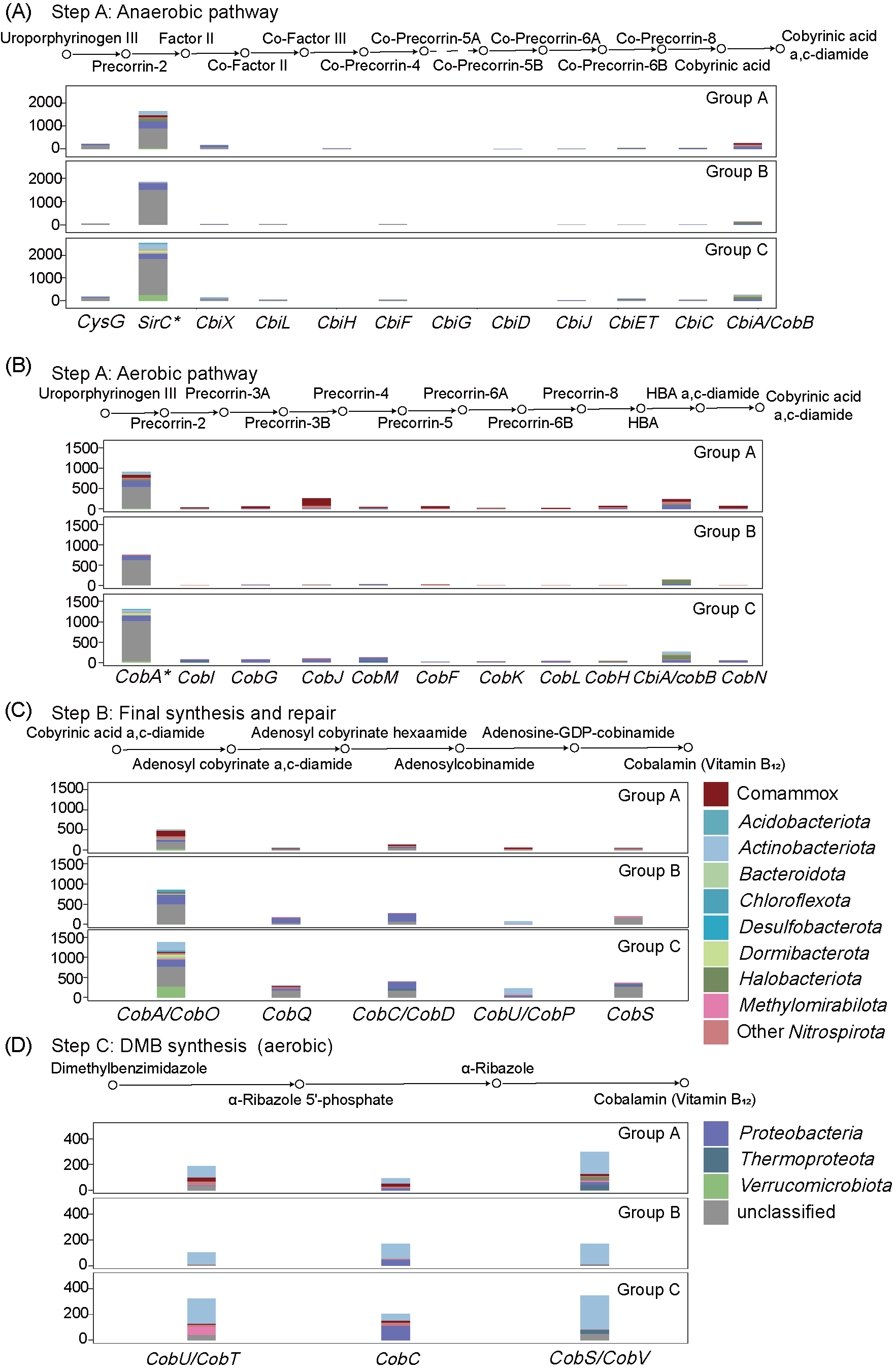


**Figure S22 Abundnace of cobalamin synthesis genes in contigs level.** (A) Gene for Step A - Anaerobic pathway. *SirC** indicates that this step can be carried out by three genes, including *CobA*, *CysG*, and *SirC*. (B) Gene for Step A - Aerobic pathway. *CobA** indicates that this step can be carried out by three genes, including *cobA*, *hemX*和*cobA*-*hemD.* (C). Gene for Step B: Final synthesis and repair. (D) Step C: DMB synthesis (aerobic).


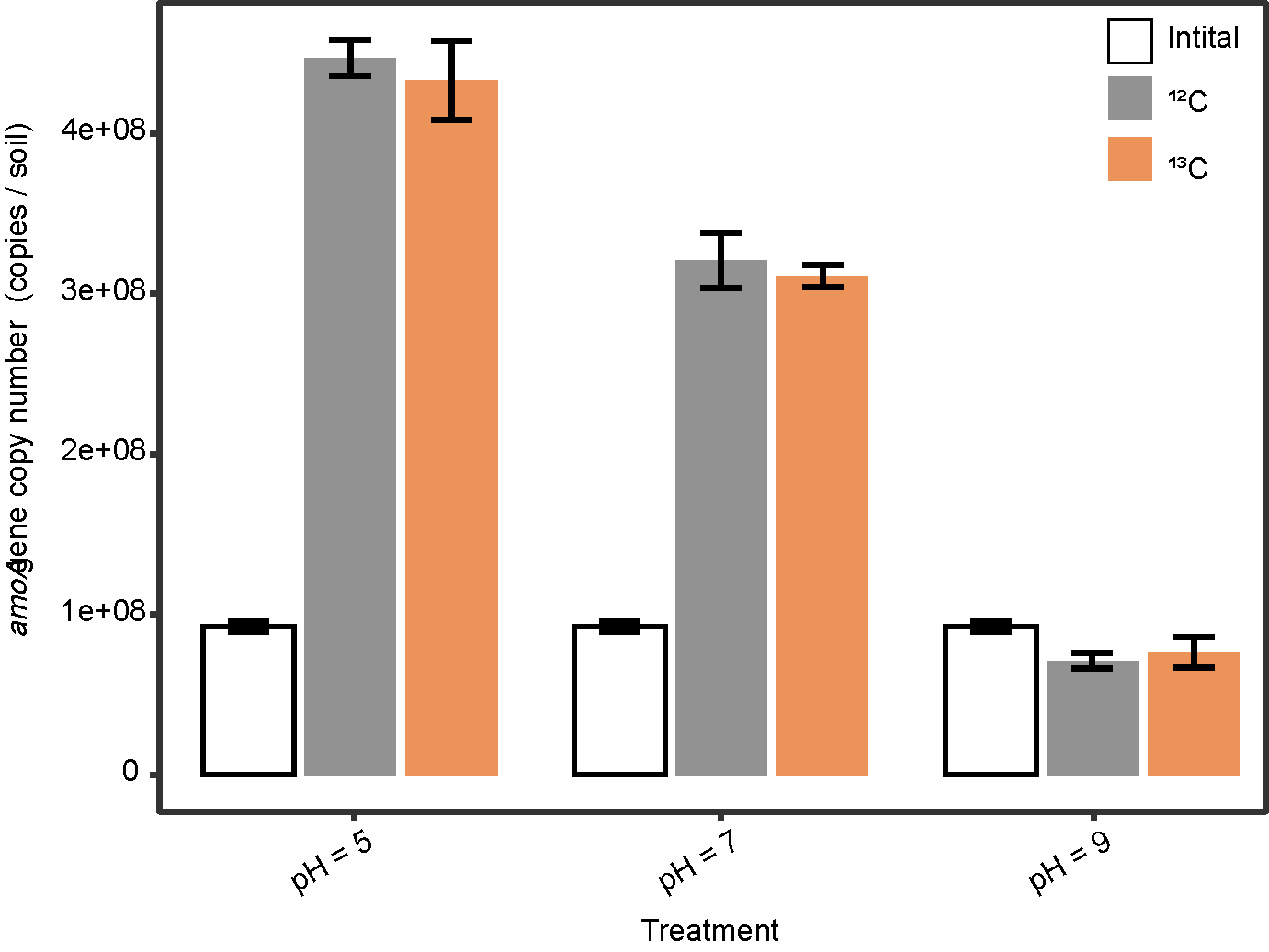


**Figure S23 The comammox *Nitropira amoA* gene copy number**. Bar graph represents the mean ± standard deviation (SD), with error bars indicating the SD.


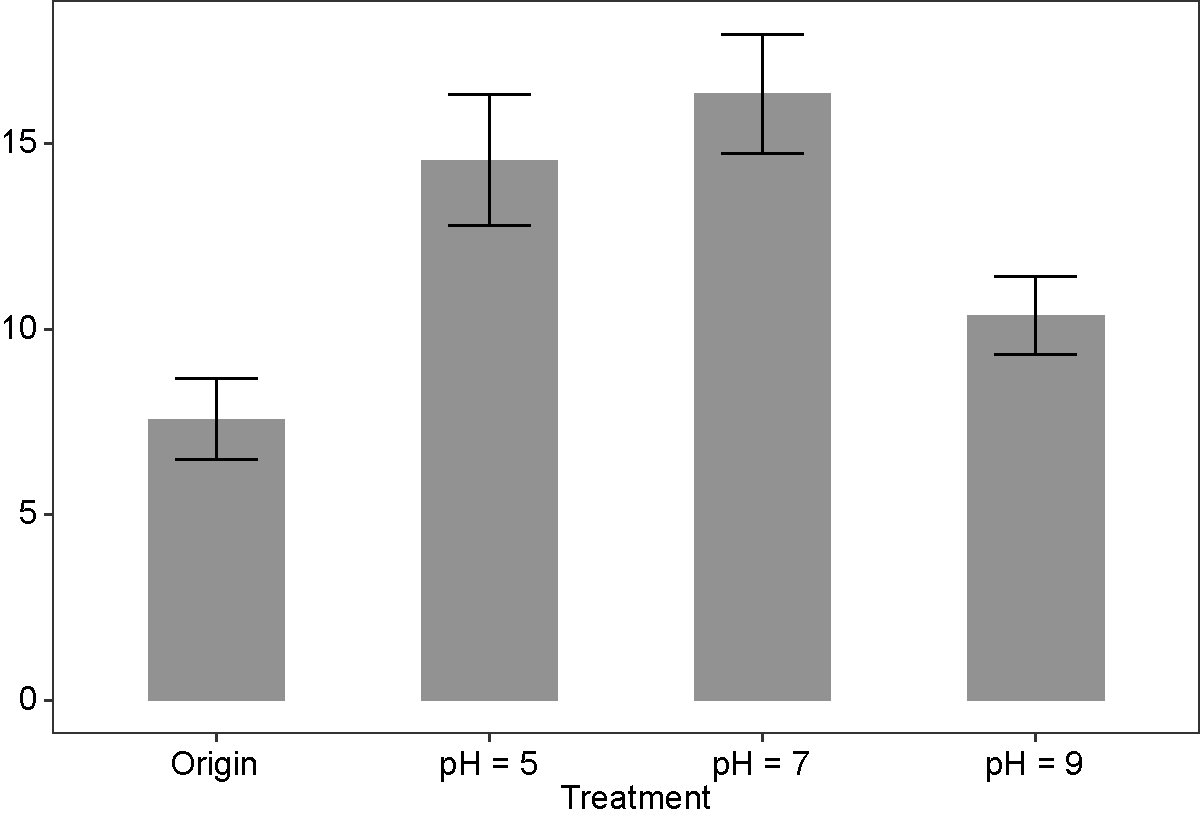


**Figure S24 Nitrification rate in different groups**. Bar graph represents the mean ± SD, with error bars indicating the SD.


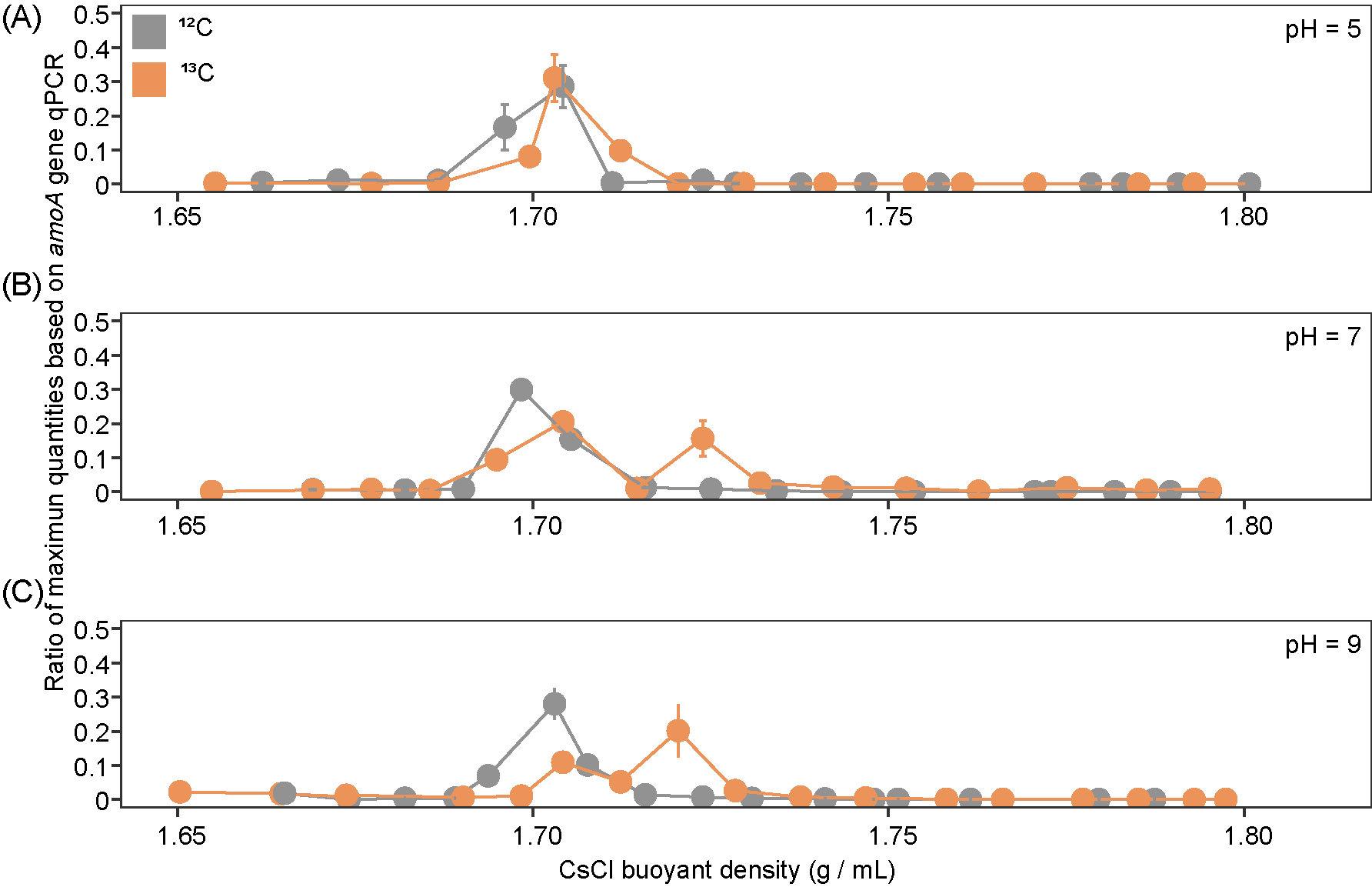


**Figure S25 CsCl buoyant density for AOB.** (A) pH = 5. (B) pH = 7. (C) pH = 9. Line graph represents the mean ± SD, with error bars indicating the SD.


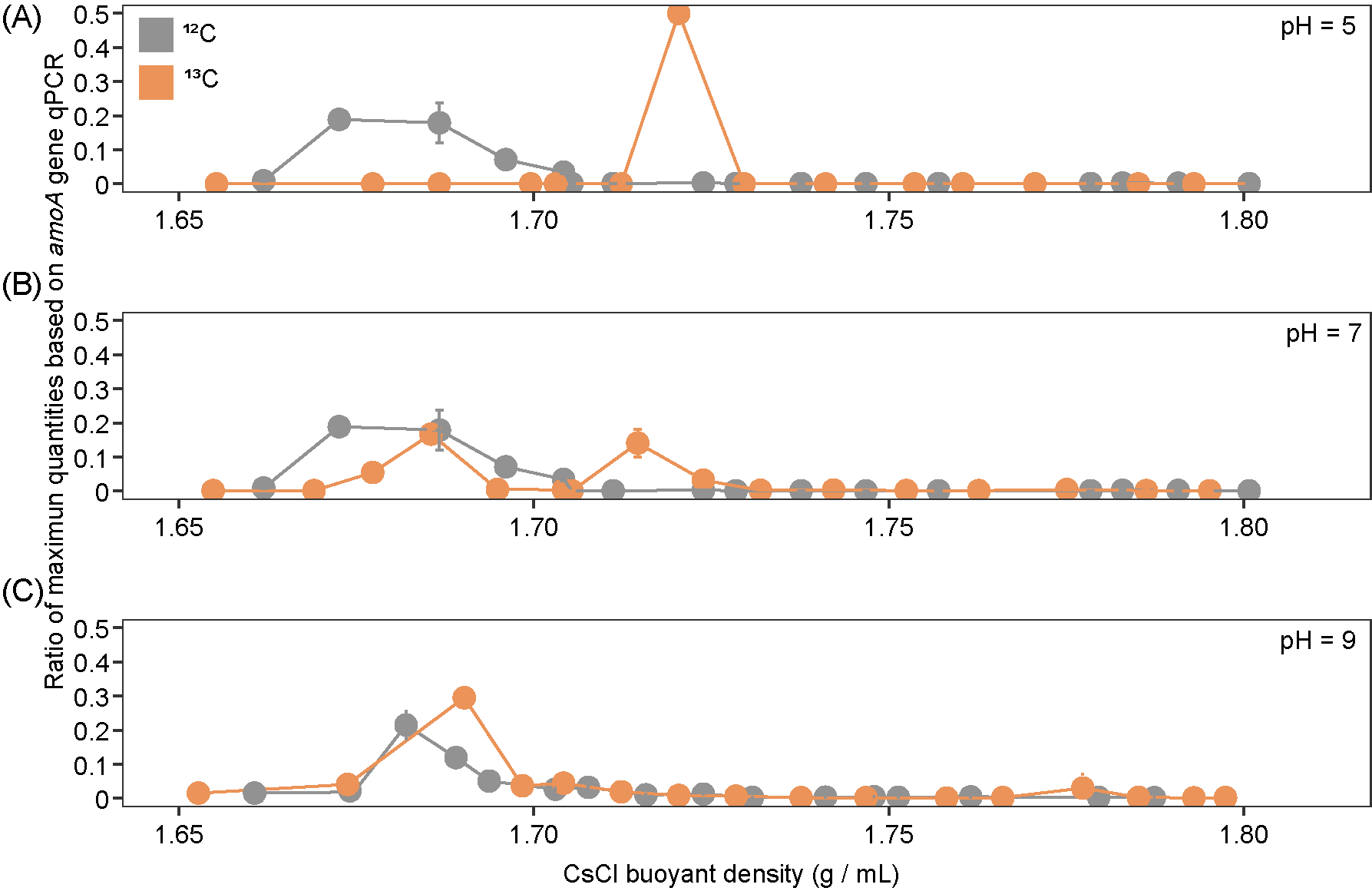


**Figure S26 CsCl buoyant density for AOA.** (A) pH = 5. (B) pH = 7. (C) pH = 9. Line graph represents the mean ± SD, with error bars indicating the SD.


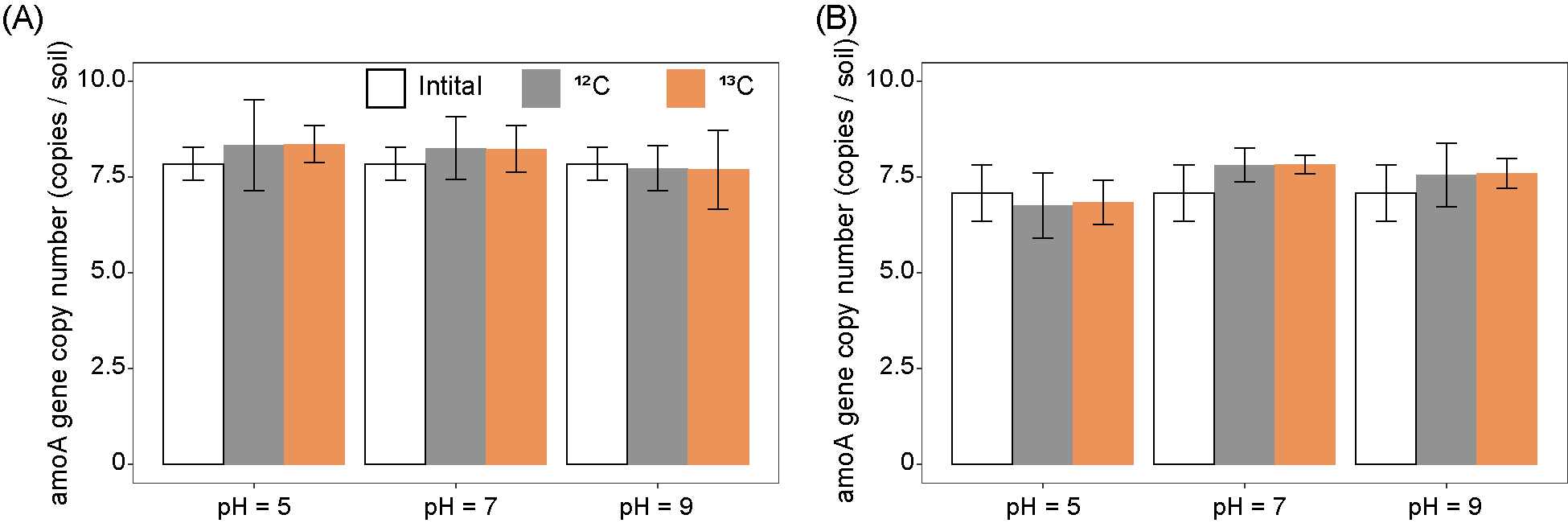


**Figure S27 *amoA* gene copy number for AOA and AOB.** (A) AOA. (B) AOB. Bar graph represents the mean ± SD, with error bars indicating the SD.


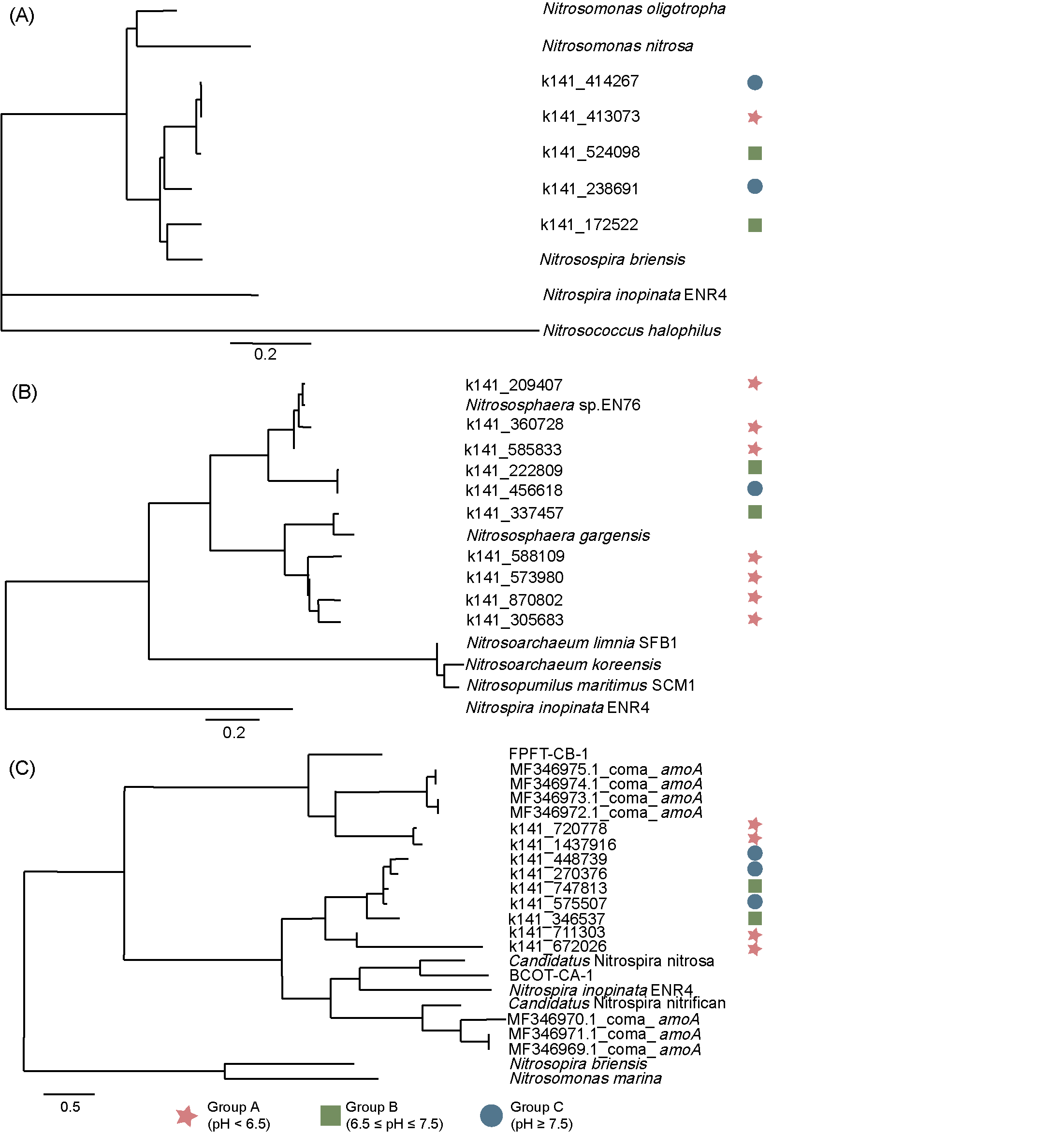


**Figure S28 Phylogenetic tree of active ammonia oxidizers.** (A) Phylogenetic trees of active AOB. (B) Phylogenetic trees of active AOA. (C) Phylogenetic trees of active comammox *Nitrospira*.


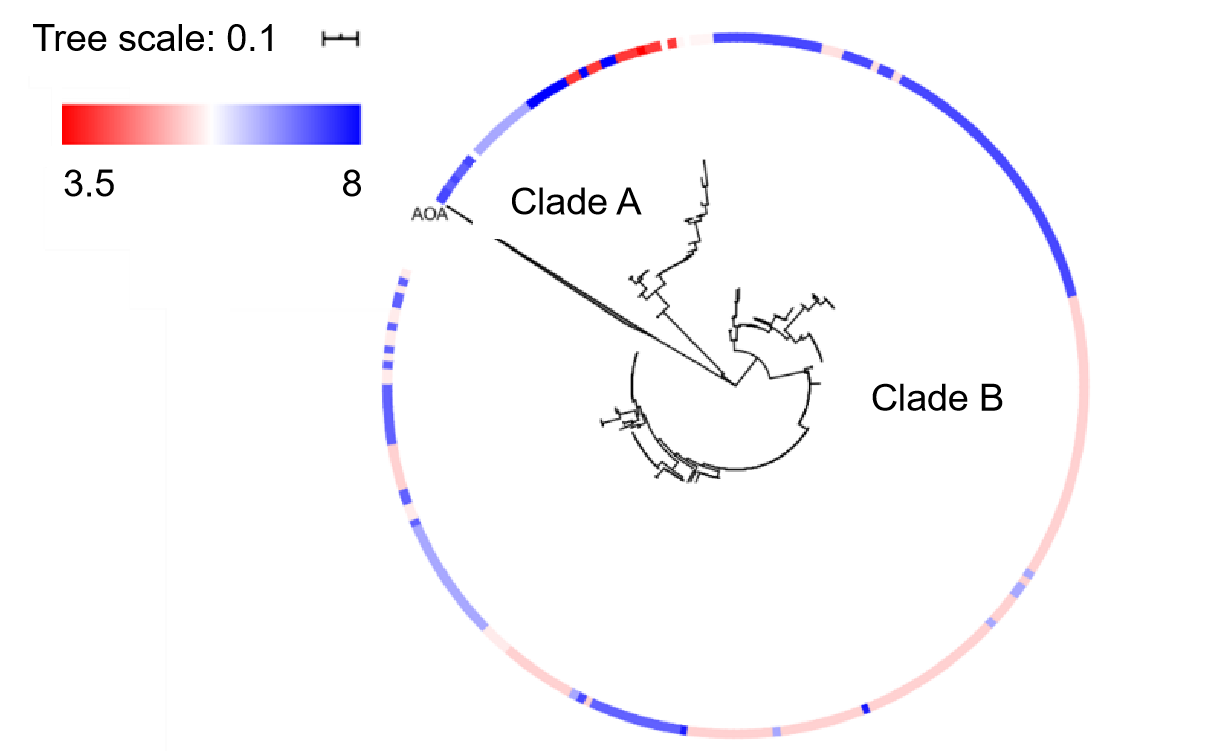


**Figure S29 Phylogeny of soil comammox *amoA* genes and their corresponding pH.** Sequences and its corresponding pH were downloaded from NCBI database.
